# SUMOylation-driven nuclear translocation of HSF2BP alleviates MASLD via COX6A1-dependent mitochondrial reprogramming

**DOI:** 10.1038/s41419-026-08935-3

**Published:** 2026-06-08

**Authors:** Mengzhou Wang, Xiaoning Wu, Tao Wang, Wuming Liu, Yuanyuan Zhang, Lin Zhang, Junzhou Zhao, Guozhi Yin, Wei Yang, Zheng Wu, Yi Lyu, Rongqian Wu

**Affiliations:** 1 Department of Hepatobiliary Surgery, The First Affiliated Hospital of Xi’an Jiaotong University, Xi’an, China; 2 National Local Joint Engineering Research Center for Precision Surgery & Regenerative Medicine, Shaanxi Provincial Center for Regenerative Medicine and Surgical Engineering, The First Affiliated Hospital of Xi’an Jiaotong University, Xi’an, China; 3 Department of Pediatrics, The First Affiliated Hospital of Xi’an Jiaotong University, Xi’an, China

**Keywords:** Non-alcoholic fatty liver disease, Sumoylation, Mechanisms of disease

## Abstract

Metabolic dysfunction-associated steatotic liver disease (MASLD) remains a major global health burden with limited therapeutic options. Heat shock factor 2 binding protein (HSF2BP), originally characterized as a germ cell-specific regulator of meiosis, is significantly upregulated in both MASLD patient livers and high-fat diet (HFD)-fed mice. Here, we identify HSF2BP as a key metabolic regulator in hepatocytes that alleviates hepatic lipid accumulation by enhancing mitochondrial function. Hepatocyte-specific overexpression of HSF2BP improves glucose tolerance, reduces lipid deposition, and increases mitochondrial respiration, whereas its knockout exacerbates steatosis. Mechanistically, we show that HSF2BP undergoes SUMOylation through interaction with UBC9, promoting its nuclear translocation and triggering an upregulation of COX6A1, a core subunit of mitochondrial complex IV. This process is impaired in MASLD due to global suppression of hepatic SUMOylation. Pharmacological inhibition of SUMOylation using TAK-981 abolishes the protective effect of HSF2BP against hepatic steatosis, whereas enhancing SUMOylation through UBC9 overexpression or treatment with the SUMO activator N106 markedly ameliorates lipid accumulation in the liver. Collectively, our findings uncover a SUMOylation-dependent mechanism by which HSF2BP regulates mitochondrial integrity and lipid homeostasis, providing a promising therapeutic axis for MASLD.

**Figure:**
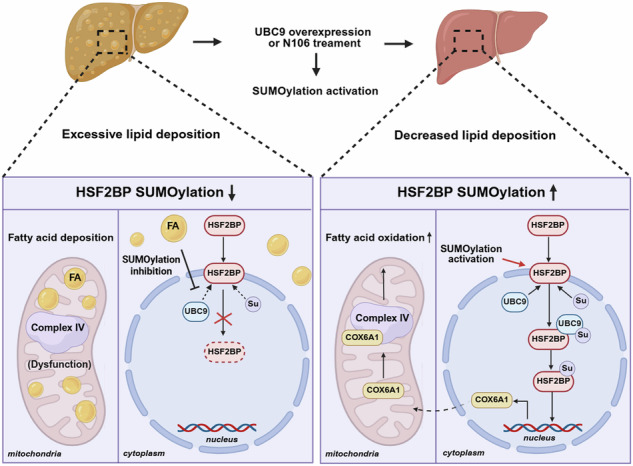

## Introduction

Metabolic dysfunction-associated steatotic liver disease (MASLD), often referred to as non-alcoholic fatty liver disease (NAFLD), is a condition characterized by the accumulation of fat in the liver due to metabolic imbalances [1]. MASLD is a complex and multifaceted disease that poses a significant global health burden. Its increasing prevalence, associated health risks, and economic impact highlight the need for continued research and improved management strategies [2, 3].

Mitochondrial dysfunction, characterized by impaired oxidative phosphorylation and increased production of reactive oxygen species (ROS), plays a critical role in the development of MASLD [4–6]. It is associated with reduced fatty acid oxidation, leading to an accumulation of lipids in the liver. This accumulation disrupts normal liver function and can trigger a cascade of events that exacerbate the disease. Additionally, the increased ROS production can induce endoplasmic reticulum (ER) stress by damaging proteins and impairing their proper folding, further contributing to the disease’s progression [7–9].

Therapies targeting mitochondrial function, such as enhancing fatty acid oxidation or reducing ROS production, are being explored as potential treatments for MASLD. By improving mitochondrial function, these interventions aim to reduce lipid accumulation and oxidative stress, thereby slowing or reversing the progression of MASLD [10–12]. Understanding the intricate relationship between mitochondrial function and MASLD is essential for developing effective strategies to manage this increasingly prevalent condition [13].

Heat shock factor 2 binding protein (HSF2BP), also known as a meiotic localizer of BRCA2 (MEILB2), was originally identified as a germ cell-specific protein that regulates meiosis in germ cells [14, 15]. Subsequent research has suggested a role for HSF2BP in cellular stress response [16, 17], meiotic homologous recombination [18, 19], and tumor progression [20–22]. Studies from our group have shown that HSF2BP is involved in acute liver injury by regulating mitochondrial function and ER stress. However, the specific role of HSF2BP in MASLD remains largely unclear. The aim of this study, therefore, was to investigate the possible role and mechanism of HSF2BP in the development of MASLD.

## Results

### HSF2BP is involved in lipid metabolism in hepatocytes

Our previous investigations have demonstrated that HSF2BP is implicated in acute liver injury [17]. To further investigate its role in hepatocytes under metabolic stress, we first generated HepG2 cell lines with HSF2BP overexpression (TG) or knockout (KO) using lentiviral vectors, along with their respective normal control (NC) cells (Fig. S1A–D). In parallel, we created hepatocyte-specific HSF2BP transgenic (TG) and knockout (HKO) mouse models via gene editing, with non-transgenic (NTG) and FLOX control animals, respectively (Fig. S1E–H).

**Fig. 1:**
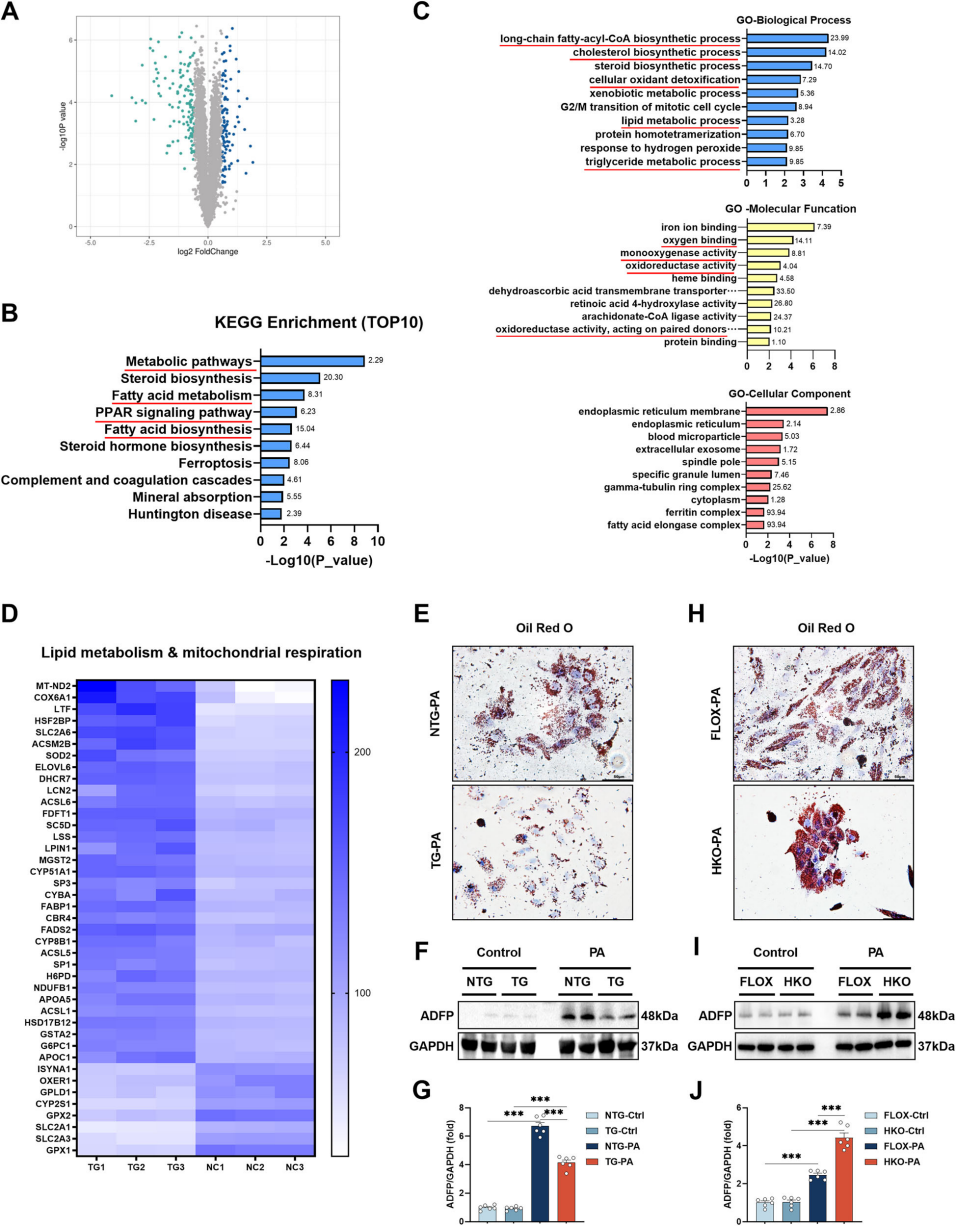
HSF2BP plays a crucial role in lipid metabolism. **A** Proteomics volcano plot of HSF2BP-overexpressing HepG2 cells; **B**, **C** proteomic analysis of HepG2 cells overexpressing HSF2BP, followed by GO and KEGG pathway enrichment analysis of differentially expressed proteins (Fold change >1.5); the numerical values adjacent to the bars represent fold enrichment; **D** proteomic analysis of differentially expressed proteins (Fold change >1.5) associated with lipid metabolism and mitochondrial respiratory chain; **E** representative images of Oil Red O staining in primary hepatocytes from HSF2BP-TG mice compared to NTG mice after 12 h of palmitic acid (PA) treatment; **F**, **G** the Western blot (WB) analysis of ADFP in primary hepatocytes from the HSF2BP-NTG group versus TG group (*n* = 6/group); **H** representative images of Oil Red O staining in primary hepatocytes from HSF2BP-HKO mice compared to FLOX mice after 12 h of PA treatment; **I**, **J** the WB analysis of ADFP in primary hepatocytes from the HSF2BP-FLOX group versus HKO group mice (*n* = 6/group). Data are presented as mean ± SEM. ∗∗∗*p* < 0.001.

We then conducted comparative quantitative proteomic analysis between HSF2BP TG and NTG cells (Fig. 1A–D). Gene Ontology (GO) and KEGG enrichment analyses revealed a strong association of HSF2BP with lipid metabolism and mitochondrial function (Fig. 1B, C). Notably, differential protein analysis showed significant upregulation of factors involved in fatty acid transport and the mitochondrial respiratory chain in TG cells (Fig. 1D).

To functionally validate these findings, we isolated primary hepatocytes from hepatocyte-specific HSF2BP TG, HKO, and respective control mice. Following 12-h treatment with palmitic acid (PA, 0.25 mM), Oil Red O staining demonstrated reduced lipid accumulation in hepatocytes from TG mice (Fig. 1E). Consistently, PA-induced upregulation of adipose differentiation-related protein (ADFP), a marker of intracellular lipid droplets, was markedly attenuated in TG-derived hepatocytes compared to NTG controls (Fig. 1F, G). Conversely, hepatocytes from HKO mice exhibited enhanced lipid deposition and increased ADFP expression relative to FLOX controls (Fig. 1H–J). Collectively, these results identify HSF2BP as a key modulator of hepatic lipid metabolism.

### HSF2BP is upregulated in the liver of patients with MASLD and high-fat diet (HFD)-fed mice

**Fig. 2:**
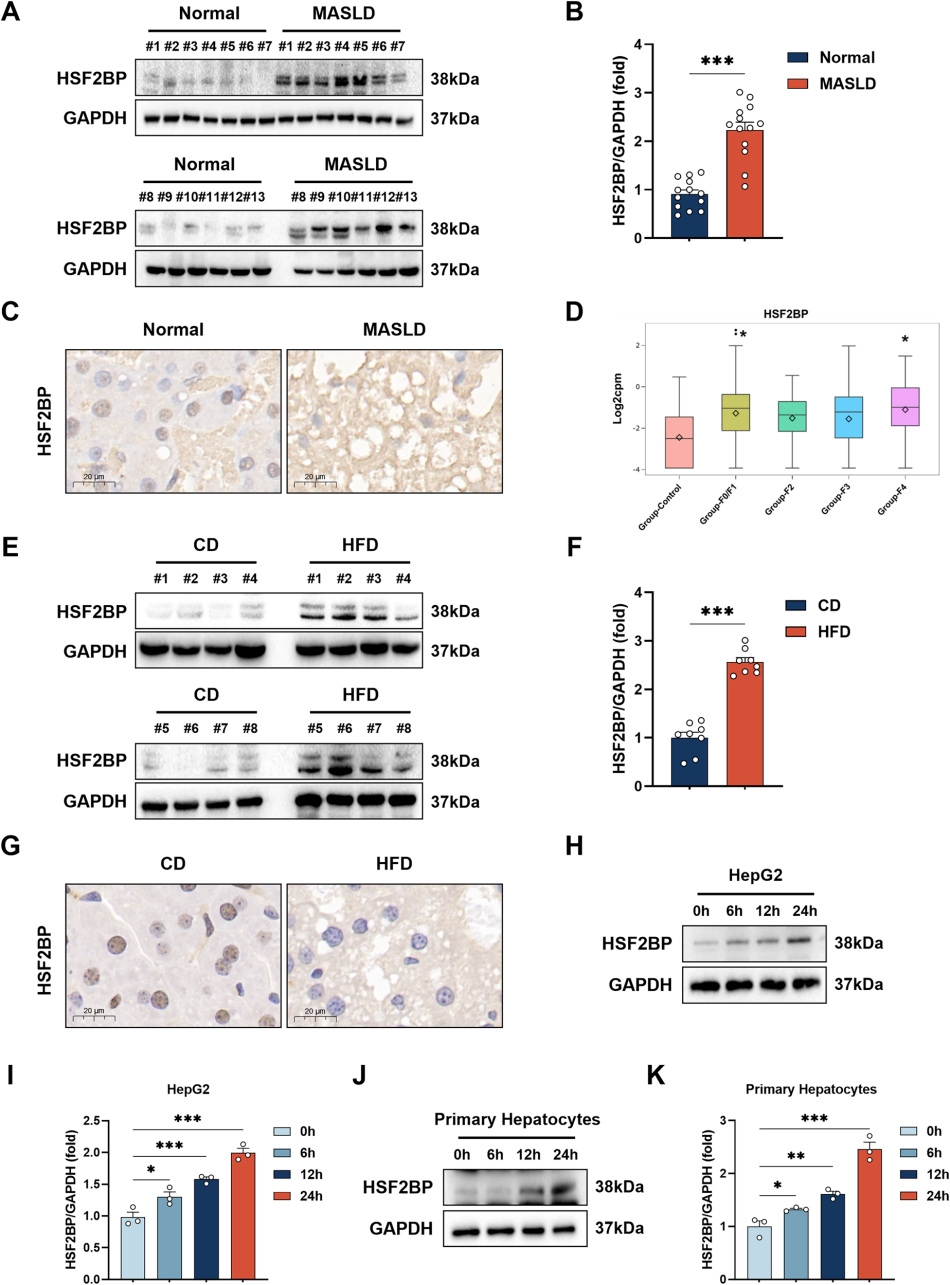
HSF2BP is upregulated in the fatty liver. **A**, **B** WB analysis of HSF2BP protein expression levels in livers of healthy individuals (*n* = 13) and patients with MASLD (*n* = 13); **C** representative immunohistochemical staining of HSF2BP in liver sections of healthy individuals and MASLD patients; **D** HSF2BP gene expression levels in the SteatoSITE database across clinically normal controls and MASLD patients at progressively advanced stages of disease severity (F0–F4). **E**, **F** WB analysis of HSF2BP protein expression levels in livers of chow diet-fed mice (CD, *n* = 8) and high fat diet-fed mice (HFD, *n* = 8); **G** representative immunohistochemical staining of HSF2BP in liver sections of CD mice and high HFD mice; **H**, **I** WB analysis of HSF2BP protein expression levels in HepG2 cells after PA treatment for 0, 6, 12, 24 h (*n* = 3/group); **J**, **K** WB analysis of HSF2BP protein expression levels in primary hepatocytes from C57BL/6 mice after palmitic acid (PA) treatment for 0, 6, 12, 24 h (*n* = 3/group). Data are presented as mean ± SEM. ∗*p* < 0.05, ∗∗*p* < 0.01, ∗∗∗*p* < 0.001.

To explore the involvement of HSF2BP in MASLD, we first assessed its hepatic expression in clinical samples. Western blot and immunohistochemical analyses revealed significantly elevated HSF2BP protein levels in liver tissues from MASLD patients compared to healthy controls (Fig. 2A–C). Supporting this observation, interrogation of the SteatoSITE transcriptomic database, a data-sharing platform for MASLD [23], showed a consistent upregulation of HSF2BP mRNA across various disease stages, with statistically significant increases at both early (F0/F1) and advanced (F4) fibrosis stages (Fig. 2D).

In parallel, wild-type mice fed a high-fat diet (HFD) for 16 weeks exhibited markedly increased hepatic HSF2BP expression compared to chow-fed controls, as demonstrated by immunoblotting and immunostaining (Fig. 2E–G). To further assess whether lipotoxic stress directly regulates HSF2BP, we treated primary mouse hepatocytes and HepG2 cells with PA (0.25 mM). PA exposure progressively elevated HSF2BP protein levels in both models (Fig. 2H–K), indicating that lipotoxicity is a potent upstream stimulus of HSF2BP induction.

### HSF2BP overexpression attenuates hepatic steatosis in HFD-fed mice

**Fig. 3:**
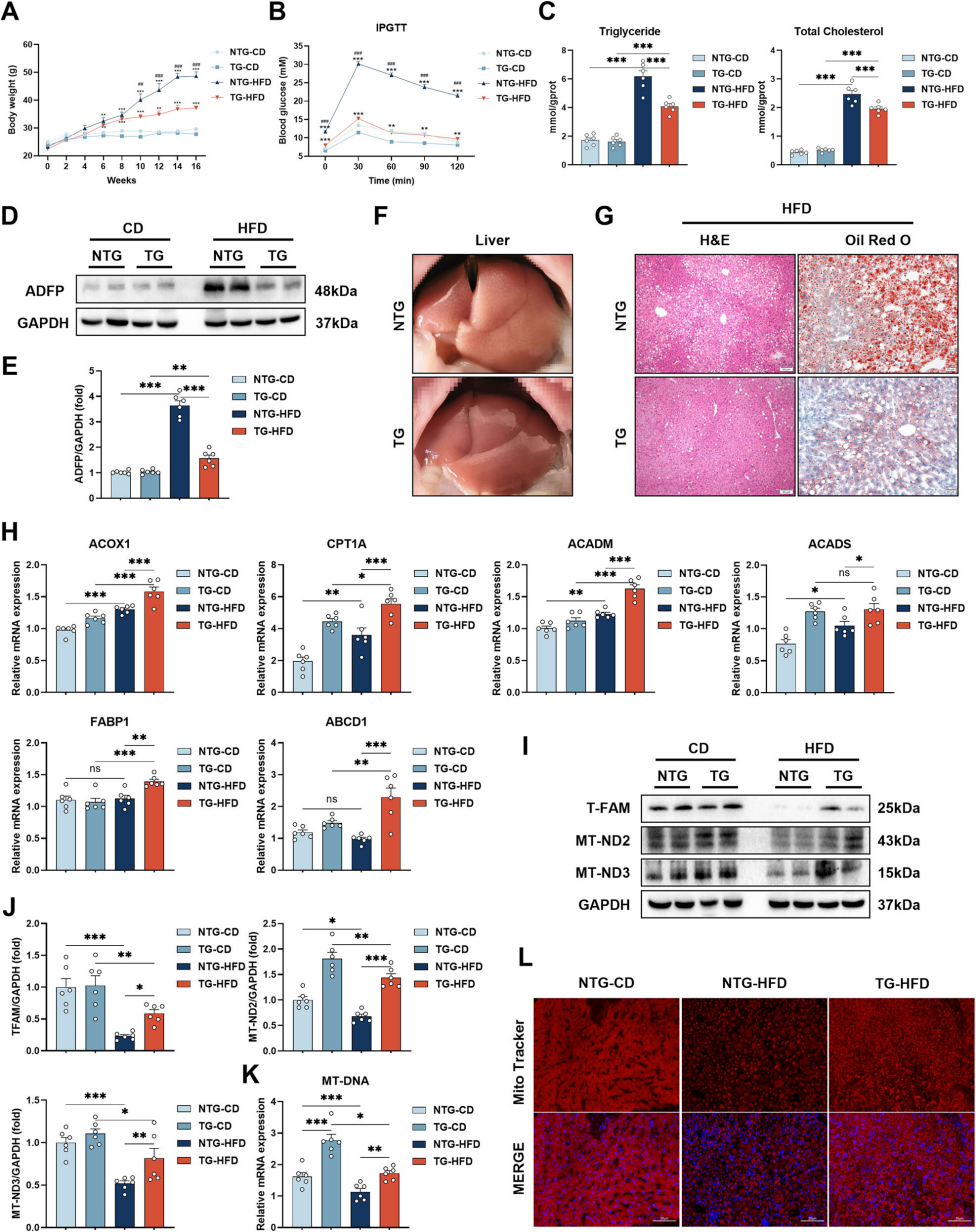
HSF2BP alleviates hepatic lipid deposition and oxidative damage in mice. Changes in body weight (**A**) and IPGTT analysis (**B**) of HSF2BP-TG and NTG groups of C57 mice fed with CD or HFD for 16 weeks (*n* = 6–8/group); * (CD vs. HFD); ^#^ (NTG-HFD vs. TG-HFD); **C** changes in hepatic triglyceride/total cholesterol content of HSF2BP-TG and NTG groups (*n* = 6/group); **D**, **E** Western blot analysis of ADFP protein expression levels in livers of C57 NTG and HSF2BP-TG mice (*n* = 6/group); **F** the liver phenotype of C57 mice in NTG and HSF2BP-TG groups fed with HFD for 16 weeks; **G** HE and Oil Red O staining of livers from C57 NTG and HSF2BP-TG mice fed HFD for 16 weeks; **H** qPCR analysis of mRNA expression levels of hepatic β-oxidation related genes (ACOX1, COX6A1, CPT1a, ACADM, ACADS, FABP1, ABCD1) in C57 NTG and HSF2BP-TG mice (*n* = 6/group); **I**, **J** WB analysis of mitochondrial-related proteins (TFAM, MT-ND2, MT-ND3) expression levels in livers of C57 NTG and HSF2BP-TG mice (*n* = 6/group); **K** the expression level of mitochondrial marker MT-ND1 mRNA in the liver of HSF2BP-TG and NTG mice (*n* = 6/group); **L** representative images of mitochondrial Tracker staining in liver tissues of C57 NTG and HSF2BP-TG mice fed HFD for 16 weeks; Data are presented as mean ± SEM. ∗*p* < 0.05, ∗∗*p* < 0.01, ∗∗∗*p* < 0.001; ###*p* < 0.001.

To evaluate the functional role of HSF2BP in hepatic steatosis in vivo, we utilized hepatocyte-specific HSF2BP TG and NTG mice fed either a chow diet (CD) or HFD for 16 weeks. Compared to HFD-NTG controls, HFD-TG mice exhibited a significant reduction in body weight beginning at week 10 (Fig. 3A), along with improved fasting blood glucose levels and enhanced glucose tolerance (Fig. 3B). Hepatic triglyceride and total cholesterol levels were markedly decreased in the HFD-TG group (Fig. 3C), accompanied by reduced protein levels of adipose ADFP (Fig. 3D, E). Macroscopically, livers from HFD-TG mice appeared dark red, in contrast to the yellowish steatotic livers observed in NTG mice (Fig. 3F). Histological staining confirmed that HSF2BP overexpression significantly ameliorated hepatic lipid accumulation (Fig. 3G). Furthermore, mRNA expression of genes involved in fatty acid β-oxidation—including acyl-CoA oxidase 1 (ACOX1), cytochrome c oxidase subunit 6A1 (COX6A1), carnitine palmitoyltransferase 1A (CPT1a), medium-chain acyl-CoA dehydrogenase (ACADM), short-chain acyl-CoA dehydrogenase (ACADS), fatty acid-binding protein 1 (FABP1), and ATP-binding cassette subfamily D member 1 (ABCD1)—was significantly upregulated in TG livers (Fig. 3H).

In contrast, HSF2BP HKO mice displayed the opposite phenotype under HFD feeding, with increased body weight (Fig. S2A), worsened glucose tolerance (Fig. S2B), and elevated hepatic lipid deposition (Fig. S2C–G) compared to FLOX controls. Genes associated with β-oxidation were downregulated in HFD-HKO livers, including ACOX1, COX6A1, CPT1a, ACADM, ACADS, FABP1, and ABCD1 (Fig. S2H). Given that mitochondrial dysfunction is a key contributor to hepatic steatosis, we next assessed mitochondrial parameters. Overexpression of HSF2BP upregulated the protein levels of mitochondrial transcription factor A (TFAM) and mitochondrially encoded NADH dehydrogenase 2 and 3 (MT-ND2, MT-ND3) (Fig. 3I, J), while HFD-fed NTG mice exhibited downregulation of these proteins. Moreover, mitochondrial content (MT-ND1) and MitoTracker staining were increased in TG livers and decreased in NTG controls (Fig. 3K, L). HSF2BP deletion further impaired mitochondrial function, reducing TFAM, MT-ND2/ND3 expression (Fig. S2I, J), mitochondrial content (Fig. S2K), and mitochondrial mass (Fig. S2L).

We next investigated the impact of HSF2BP on endoplasmic reticulum (ER) stress and oxidative stress, two interconnected contributors to lipotoxic liver injury. HFD feeding induced significant upregulation of ER stress markers in NTG mice—including phosphorylated inositol-requiring enzyme 1α (p-IRE1α), binding immunoglobulin protein (BIP), spliced X-box binding protein 1 (XBP1s), and C/EBP homologous protein (CHOP)—while HSF2BP overexpression markedly attenuated their expression (Fig. S3A, B). In addition, the antioxidant enzyme superoxide dismutase 2 (SOD2) was elevated in TG mice (Fig. S3C, D), and dihydroethidium (DHE) staining revealed reduced reactive oxygen species (ROS) accumulation (Fig. S3E). Biochemical assays confirmed decreased levels of malondialdehyde (MDA), a lipid peroxidation marker, and increased levels of antioxidants glutathione (GSH) and total superoxide dismutase (SOD) in HFD-TG livers (Fig. S3F–H).

Conversely, HSF2BP deficiency exacerbated oxidative and ER stress under HFD feeding. HFD-HKO mice exhibited increased expression of BIP, XBP1s, and CHOP (Fig. S3I, J), decreased SOD2 protein levels (Fig. S3K, L), enhanced hepatic ROS production (Fig. S3M), and an oxidative stress profile marked by elevated MDA and decreased GSH and SOD levels (Fig. S3N–P). Together, these data indicate that HSF2BP protects against HFD-induced hepatic steatosis through coordinated enhancement of mitochondrial integrity and attenuation of ER and oxidative stress.

### HSF2BP SUMOylation regulates its subcellular localization and function

**Fig. 4:**
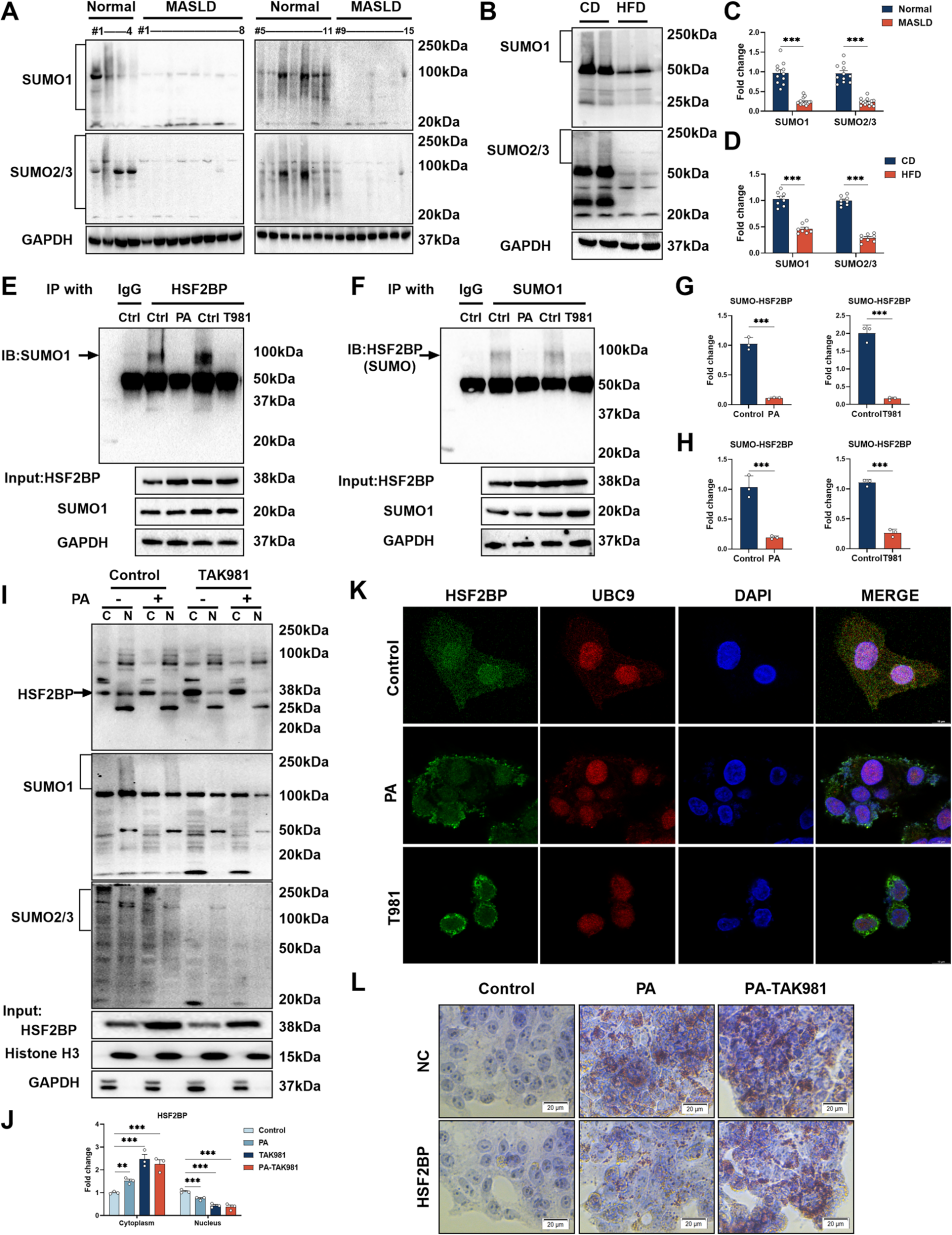
SUMOylation of HSF2BP affects its cellular localization and function. **A**, **C** WB analysis of SUMOylation protein levels in normal human liver (*n* = 11) and MASLD patient liver tissues (*n* = 15); **B**, **D** WB analysis of SUMOylation protein levels in the liver of C57 mice under CD and HFD conditions (*n* = 6/group); **E**, **G** immunoprecipitation (IP) with anti-HSF2BP antibody in NC, PA-treated, and TAK-981-treated groups, then IB with anti-SUMO1 antibody (*n* = 3/group); **F**, **H** IP with anti-SUMO1 antibody in NC, PA-treated, and TAK-981-treated groups, then IB with anti-HSF2BP antibody (*n* = 3/group); **I**, **J** WB detection of HSF2BP expression levels in nuclear and cytoplasmic fractions of HepG2 cells treated with control or T981 after 12 h of PA treatment (*n* = 3/group); **K** representative images of subcellular localization staining of HSF2BP protein in HepG2 cells following treatments with control, PA (12 h), and T981 (48 h); **L** representative images of Oil Red O staining of HepG2 cell lines NC group and HSF2BP-TG group after control, PA (12 h), and T981 (48 h) + PA (12 h) treatments. Data are presented as mean ± SEM. ∗∗*p* < 0.01, ∗∗∗*p* < 0.001.

SUMOylation is a reversible post-translational modification that regulates diverse cellular processes, including lipid metabolism. Dysregulated SUMOylation contributes to lipid accumulation and the pathogenesis of steatotic liver diseases [24, 25]. Analysis of human liver tissues from MASLD patients revealed a global reduction in protein SUMOylation compared to healthy controls (Fig. 4A, C). We quantified the conjugated form of SUMO, represented by the characteristic high‑molecular‑weight smeared bands (the “SUMOylation smear”), to assess overall hepatic or cellular SUMOylation levels, rather than the free SUMO molecules migrating at approximately 20 kDa. Consistently, transcriptomic data from the SteatoSITE database showed a stage-dependent downregulation of small ubiquitin-like modifier 1 (SUMO1) expression in MASLD progression (Fig. S4A). Similarly, wild-type (WT) mice fed an HFD for 16 weeks exhibited decreased hepatic SUMOylation compared to CD controls (Fig. 4B, D).

SUMOylation is known to modulate protein subcellular localization [26]. Our previous immunohistochemistry results indicated that HSF2BP translocates from the nucleus to the cytoplasm in both human and mouse fatty liver specimens (Fig. 2C, G). To investigate whether SUMOylation regulates the subcellular localization and function of HSF2BP, we first treated HepG2 cells with palmitic acid (PA, 0.25 mM, 12 h) or the selective SUMO E1 inhibitor TAK-981 (0.1 μM, 48 h) [27–30], and we validated the inhibitory effect of TAK-981 on cellular SUMOylation using a dose-response experiment in HepG2 cells with a 48-h treatment (Fig. S4B, C).

In lysates treated with the de-SUMOylation inhibitor NEM, immunoprecipitation (IP) using anti-HSF2BP followed by immunoblotting (IB) revealed an additional band at ~75–100 kDa, in addition to the expected 38 kDa band. This higher-molecular-weight band was markedly reduced upon PA treatment or SUMOylation inhibition by TAK-981 (Fig. S4C, D). Further IP/IB analyses showed that this band was detected specifically with anti-SUMO1, but not anti-SUMO2/3 (Fig. 4E, G). Reciprocal co-immunoprecipitation using anti-SUMO1 followed by anti-HSF2BP IB confirmed the same pattern (Fig. 4F, H). Moreover, overexpression of V5-tagged SUMO1, but not SUMO2/3, enhanced the intensity of the ~75–100 kDa band, which was abolished by TAK-981 (Fig. S5A–D). In addition, this band was found to interact with UBC9 (Fig. S5E, F), further supporting its involvement in the SUMOylation pathway. Therefore, the 75–100 kDa band represents SUMOylated HSF2BP, demonstrating that HSF2BP can be modified by SUMO1, which is consistent with previous reports [31].

Using online prediction tools, we identified two candidate lysine residues, K75 and K222. Mutagenesis revealed that K222R (but not K75R) substantially reduced HSF2BP SUMOylation (Fig. S6C, D). Fluorescence staining showed that the K222R mutation altered HSF2BP subcellular localization (Fig. S6E). These findings indicate that K222 might serve as a major SUMOylation site of HSF2BP.

Nuclear‑cytoplasmic fractionation and WB analysis confirmed that PA or TAK-981 treatment reduced nuclear HSF2BP while increasing its cytoplasmic levels (Fig. 4I, J). Interestingly, we found that SUMO1 is predominantly nuclear, while SUMO2/3 is present in both the cytoplasm and nucleus, with a higher proportion in the cytoplasm. Consequently, quantification of the high-molecular-weight smear was performed specifically in the nuclear fraction for SUMO1, and in both the nuclear and cytoplasmic fractions for SUMO2/3. Immunofluorescence staining further supported the PA‑ or TAK‑981‑induced nuclear‑to‑cytoplasmic relocalization of HSF2BP (Fig. 4K). Functionally, TAK-981 abolished the protective effects of HSF2BP overexpression against PA-induced lipid accumulation (Fig. 4L), reactive oxygen species (ROS) production (Fig. S7A), and mitochondrial dysfunction (Fig. S7B–H).

**Fig. 5:**
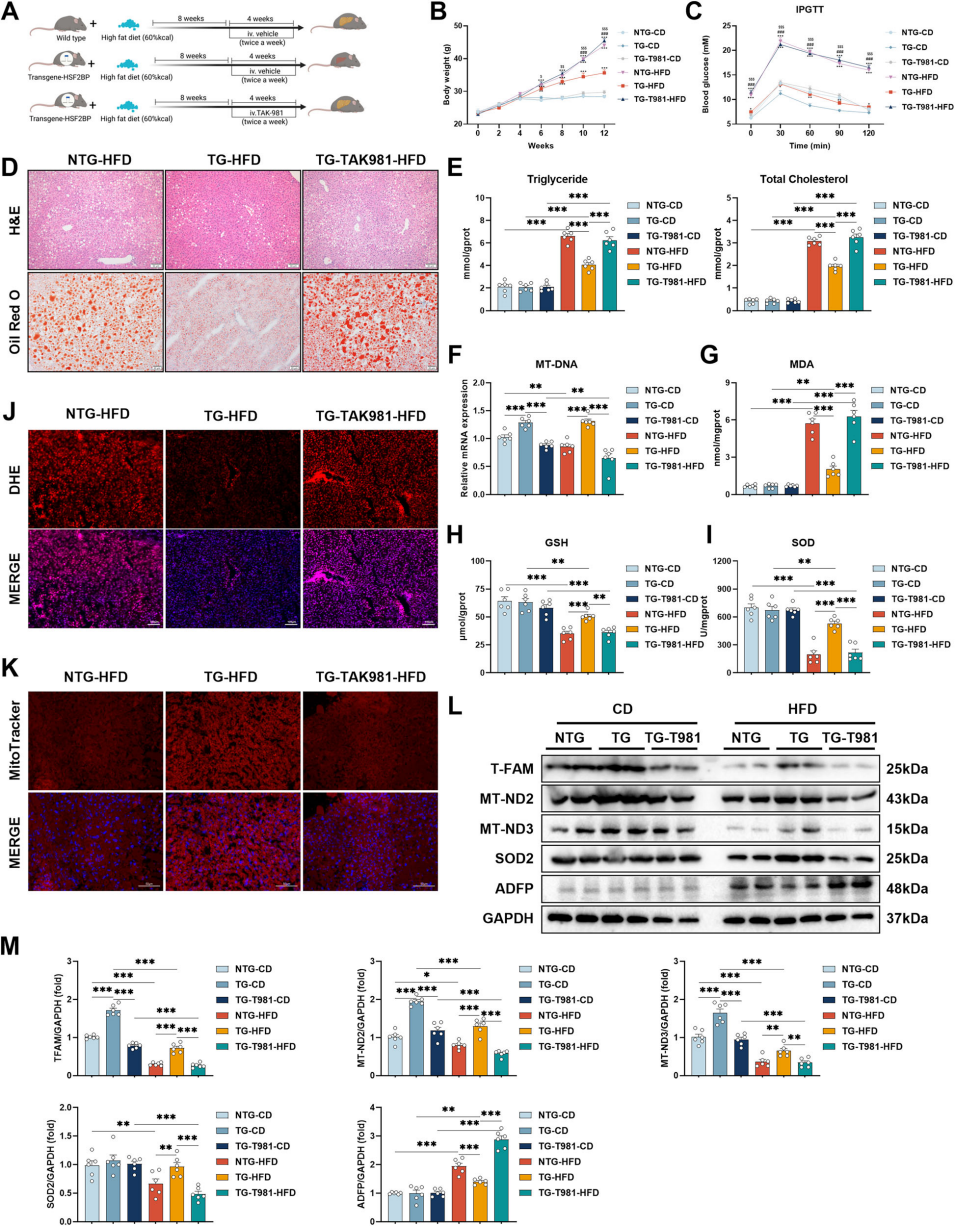
Animal experiments validate loss of lipid regulatory function upon inhibition of HSF2BP SUMOylation. **A** Flowchart of animal experimentation procedure; changes in body weight (**B**) and IPGTT analysis (**C**) of C57 mice fed with CD or HFD diets for 12 weeks in NTG, TG, and TG-T981 groups (*n* = 6/group); * (CD vs. HFD); ^#^ (NTG-HFD vs. TG-HFD); ^$^(TG-HFD vs. TG-HFD); **D** liver HE and Oil Red O staining of C57 mice NTG, HSF2BP-TG, and HSF2BP-TG+T981 groups fed with HFD diet for 12 weeks; **E** liver triglyceride and cholesterol content in C57 mice NTG, HSF2BP-TG, and HSF2BP-TG+T981 groups fed with CD or HFD diets for 12 weeks (*n* = 6/group); **F** mRNA expression levels of mitochondrial marker gene MT-ND1 in liver tissue of C57 mice NTG, HSF2BP-TG, and HSF2BP-TG+T981 groups (*n* = 6/group); **G**–**I** liver tissue MDA, GSH and SOD content in C57 mice NTG, HSF2BP-TG, and HSF2BP-TG+T981 groups fed with HFD diet for 12 weeks (*n* = 6/group); **J** representative images of ROS staining in liver tissue of C57 mice NTG, HSF2BP-TG, and HSF2BP-TG+T981 groups fed with HFD diet for 12 weeks; **K** representative images of mitochondrial Tracker staining in liver tissue of C57 mice NTG, HSF2BP-TG, and HSF2BP-TG+T981 groups fed with HFD diet for 12 weeks; **L**, **M** WB analysis of liver ADFP, SOD2, and mitochondrial-related proteins (TFAM, MT-ND2, MT-ND3) in C57 mice NTG, HSF2BP-TG, and HSF2BP-TG+T981 groups fed with HFD diet for 12 weeks (*n* = 6/group). Data are presented as mean ± SEM. ∗*p* < 0.05, ∗∗*p* < 0.01, ∗∗∗*p* < 0.001; ###*p* < 0.001; $*p* < 0.05, $$*p* < 0.01, $$$*p* < 0.001.

In the in vivo experiments, we administered TAK-981 (7.5 mg/kg, every 3 days for 4 weeks) via tail vein injection to HFD-fed HSF2BP TG mice. Overexpression of HSF2BP significantly improved body weight, fasting blood glucose, glucose tolerance, and hepatic lipid deposition in HFD-fed mice; however, these beneficial effects were abolished upon TAK-981 treatment (Fig. 5A–E). Similarly, HSF2BP overexpression ameliorated oxidative stress and mitochondrial function in HFD-fed mice, and these improvements were likewise eliminated by TAK-981 administration (Fig. 5F–M). These results demonstrate that the protective effects of HSF2BP on hepatic lipid deposition, oxidative stress, and mitochondrial function are primarily dependent on its SUMOylated form.

Mechanistically, HSF2BP undergoes SUMOylation through interaction with UBC9, the sole SUMO E2 ligase [31]. In HepG2 cells, prolonged PA exposure led to progressive downregulation of UBC9 and global SUMOylation levels (Fig. S8A). To counteract this, we overexpressed UBC9 or treated cells with N106, a small-molecule SUMO E1 enzyme activator [32, 33]. Both UBC9 overexpression and N106 treatment (10 μM, 24 h) enhanced nuclear localization of HSF2BP (Fig. S8B) and increased SUMOylated-HSF2BP levels, as shown by immunoprecipitation (Fig. S8C). These interventions also reduced lipid accumulation in PA-treated HepG2 cells; however, this effect was abrogated upon HSF2BP knockout (Fig. S9A, B), confirming that HSF2BP is a critical effector downstream of SUMOylation.

We further validated these findings in vivo. HFD feeding for 12 weeks significantly reduced hepatic UBC9 expression in WT mice (Fig. S9C), correlating with decreased global SUMOylation. To restore SUMOylation, we delivered UBC9 via adeno-associated virus (AAV) vector through tail vein injection. Two weeks post-injection, mice were subjected to either HFD or chow diet for 12 weeks. In parallel, another group of WT mice received intraperitoneal N106 injections (10 mg/kg/day) from week 8 to week 12 of HFD feeding. Both interventions—UBC9 overexpression and N106 treatment—effectively reduced hepatic steatosis in HFD-fed mice (Fig. S9D, E). Importantly, these beneficial effects were absent in HSF2BP-HKO mice, indicating that the protective role of hepatic SUMOylation in steatosis is mediated through HSF2BP.

### The effects of HSF2BP overexpression on hepatic steatosis are mediated via upregulation of COX6A1

**Fig. 6:**
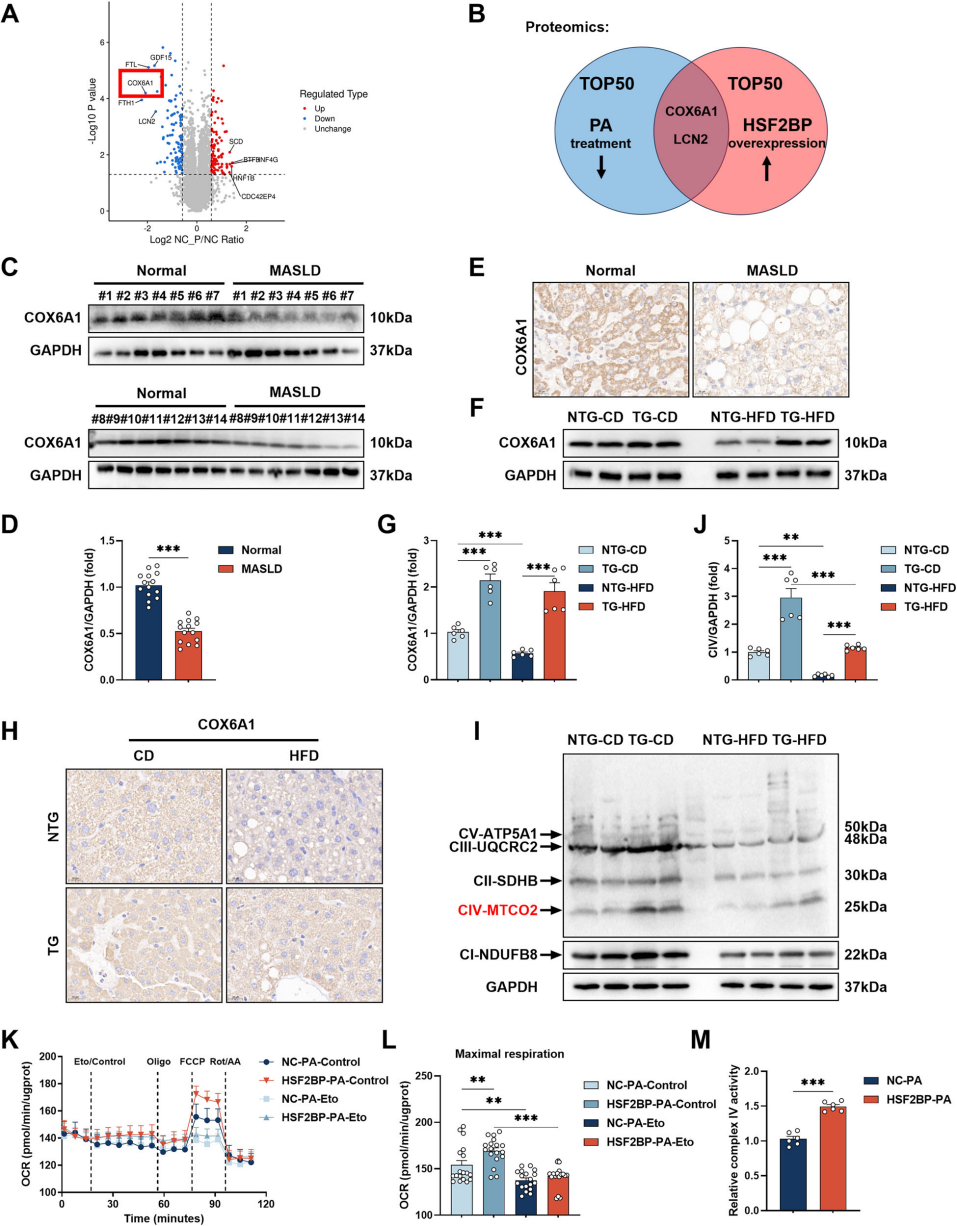
HSF2BP regulates COX6A1 and thereby affecting mitochondrial complex IV. **A** Protein profiling of HepG2 cells treated with PA for 12 h, identifying top 10 differentially expressed proteins (Fold change > 1.5); **B** the top 50 downregulated proteins induced by 12 h PA treatment in HepG2 cells showed overlap with the top 50 upregulated proteins resulting from HSF2BP overexpression; **C**, **D** WB analysis of COX6A1 protein expression in normal human liver and MASLD patient liver samples (*n* = 14/group); **E** representative immunohistochemical staining of COX6A1 in normal human liver and MASLD patient liver tissues; **F**, **G** WB analysis of COX6A1 protein expression in the livers of C57 mice from NTG and HSF2BP-TG groups fed a CD or HFD for 16 weeks (*n* = 6/group); **H** representative immunohistochemical staining of COX6A1 in C57 mouse liver from NTG and HSF2BP-TG groups fed CD or HFD for 16 weeks; **I**, **J** WB analysis of mitochondrial complex-related proteins in the livers of C57 mice from NTG and HSF2BP-TG groups (*n* = 6/group); **K**, **L** OCR analysis of HepG2 cell lines NC and HSF2BP-TG groups with palmitic acid substrate. Substrates added sequentially: Eto/control, Oligo, FCCP, Rot/AA (FCCP-induced maximal respiration reflects mitochondrial capacity for fatty acid handling) (*n* = 6–8/group); **M** activity assay of mitochondrial Complex IV in HepG2 cell lines NC and HSF2BP-TG groups after 12 h of PA treatment (*n* = 6/group). Data are presented as mean ± SEM. ∗∗*p* < 0.01, ∗∗∗*p* < 0.001.

Proteomic profiling identified cytochrome c oxidase subunit 6A1 (COX6A1) as one of the most significantly upregulated proteins in HSF2BP-overexpressing HepG2 cells (Fig. 1D). Conversely, COX6A1 expression was markedly downregulated in HepG2 cells treated with PA (Fig. 6A, B). COX6A1 is a structural subunit of cytochrome c oxidase (COX), also known as mitochondrial complex IV—the terminal and rate-limiting enzyme in the electron transport chain responsible for oxidative phosphorylation (OXPHOS) [34]. Recent studies have shown that deficiency of COX6A1 not only impairs mitochondrial complex IV function, leading to hepatocyte injury, but is also closely linked to abnormal mitochondrial oxidation and lipotoxicity in MASLD [35, 36].

In liver samples from MASLD patients, both immunohistochemical staining and Western blotting revealed reduced COX6A1 protein levels (Fig. 6C–E). Transcriptomic analysis from the SteatoSITE database further confirmed a significant downregulation of COX6A1 expression at the cirrhotic (F4) stage of MASLD (Fig. S10A). In parallel, levels of multiple mitochondrial respiratory complexes were also diminished in human MASLD liver tissues (Fig. S10B, C).

To evaluate the in vivo regulation of COX6A1 by HSF2BP, we measured hepatic COX6A1 and complex IV levels in HSF2BP-TG and NTG mice fed a CD or HFD for 16 weeks. HFD feeding led to a substantial reduction in COX6A1 expression and complex IV abundance in NTG livers, while HSF2BP overexpression rescued both parameters in HFD-fed mice (Fig. 6F–J). Similarly, HSF2BP overexpression reversed PA-induced downregulation of COX6A1 in HepG2 cells (Fig. S10D, E). However, inhibition of SUMOylation with TAK-981 abolished the COX6A1 upregulation mediated by HSF2BP, both in vitro and in vivo (Fig. S10D–I), indicating that SUMOylation is required for HSF2BP’s regulatory effect on COX6A1.

To further assess mitochondrial function, we measured the oxygen consumption rate (OCR) in HepG2 cells, limiting the mitochondrial substrate to PA to mimic a steatotic environment. OCR and maximal mitochondrial respiration were significantly enhanced in HSF2BP-TG hepatocytes compared to NTG controls (Fig. 6K, L), indicating improved mitochondrial activity under HFD conditions. This was corroborated by a marked increase in complex IV enzymatic activity in HSF2BP-TG livers (Fig. 6M).

**Fig. 7:**
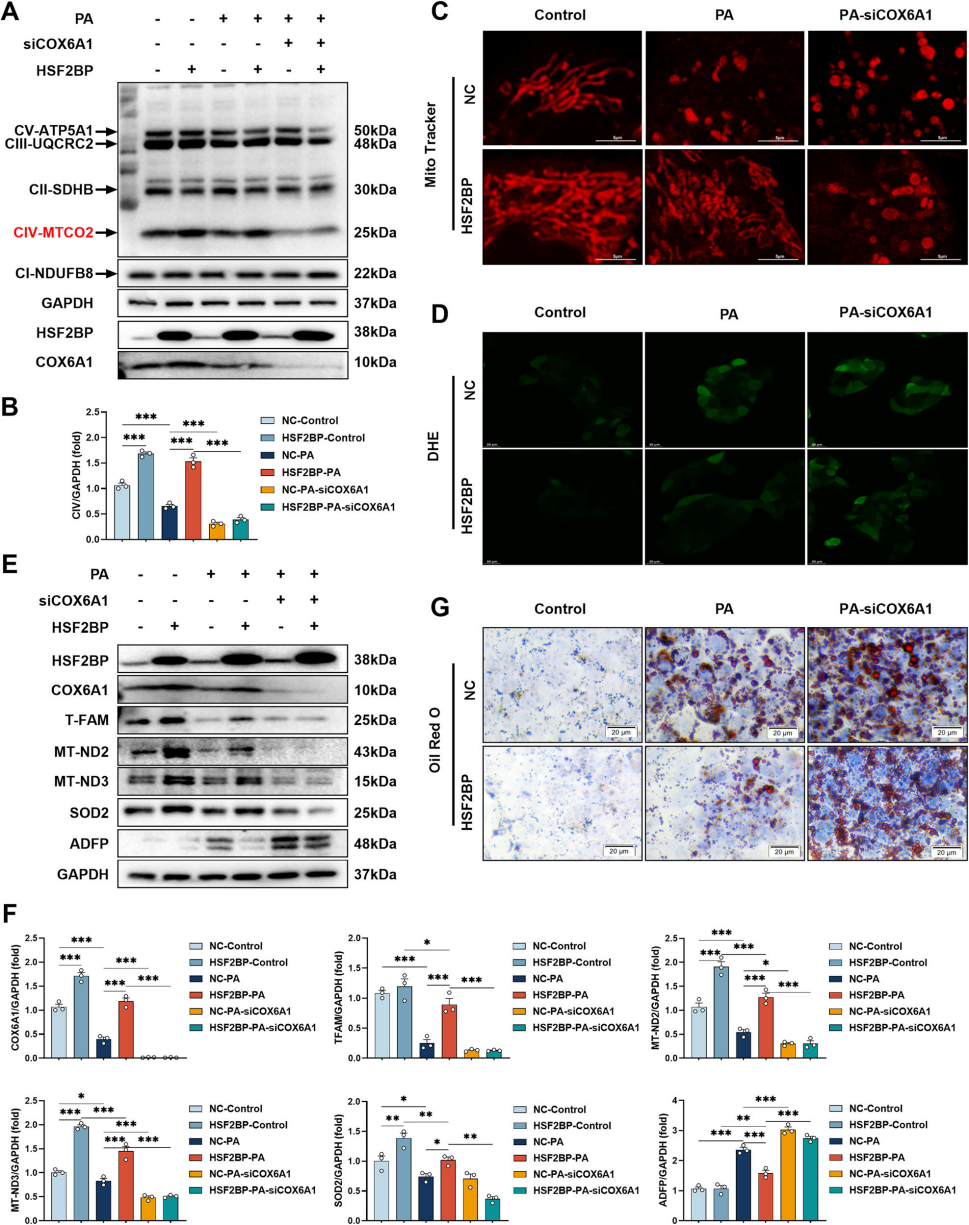
Knockout of COX6A1 abolishes the lipid and mitochondrial regulatory effects of HSF2BP. **A**, **B** WB analysis of mitochondrial complex-related proteins in NC and HSF2BP-TG HepG2 cells treated with PA for 12 h and siCOX6A1+PA (12 h) (*n* = 3/group); **C** representative images of mitochondrial Tracker staining in NC and HSF2BP-TG HepG2 cells treated as described in (**A**, **B**); **D** representative image of ROS staining in NC and HSF2BP-TG HepG2 cells treated as described in (**A**, **B**); **E**, **F** WB analysis of ADFP and mitochondrial-related proteins (TFAM, MT-ND2, MT-ND3, COX6A1) in NC and HSF2BP-TG HepG2 cells treated as described in (**A**, **B**) (*n* = 3/group); **G** representative image of Oil Red O staining in NC and HSF2BP-TG HepG2 cells treated as described in (**A**, **B**). Data are presented as mean ± SEM. ∗*p* < 0.05, ∗∗*p* < 0.01, ∗∗∗*p* < 0.001.

To establish the functional relevance of COX6A1, we overexpressed it in HepG2 cells. COX6A1 overexpression elevated complex IV levels (Fig. S11A, B), enhanced OCR and maximal respiration (Fig. S11C, D), and reduced lipid accumulation, as evidenced by Oil Red O staining (Fig. S11E) and decreased ADFP expression (Fig. S11F, G). Conversely, knockdown of COX6A1 with siRNA (siCOX6A1) abrogated the protective effects of HSF2BP overexpression. Specifically, COX6A1 knockdown suppressed complex IV induction (Fig. 7A, B), reduced mitochondrial content (Fig. 7C), increased ROS production (Fig. 7D), and exacerbated lipid accumulation (Fig. 7E–G), confirming that COX6A1 is a key downstream effector in HSF2BP-mediated mitochondrial and metabolic regulation.

## Discussion

MASLD has emerged as the most prevalent chronic liver disease worldwide and is a leading cause of liver transplantation, particularly in Western countries [37, 38]. Despite its growing global burden, effective therapeutic strategies remain limited. In this study, we identify HSF2BP as a novel regulator of hepatic lipid metabolism and mitochondrial function and demonstrate that its SUMOylation is essential for mitigating MASLD progression.

Originally characterized as a testis-specific HSF2-interacting protein [15], HSF2BP has been implicated in stress responses. However, in our chronic MASLD model, overexpression of HSF2BP did not alter HSF2 or HSP70 levels, suggesting a divergent role compared to its known function in acute liver injury [39–41]. This points to a context-dependent regulatory mechanism, in which HSF2BP may operate independently of the canonical heat shock response in chronic metabolic liver disease.

Proteomic profiling and functional studies revealed that HSF2BP regulates mitochondrial oxidative capacity and lipid clearance by upregulating COX6A1, a component pivotal to the functionality of the mitochondrial respiratory chain complex IV [34]. COX6A1 governs the final electron transfer step in oxidative phosphorylation and has recently garnered interest for its role in maintaining hepatocyte integrity under lipotoxic stress [35, 42–44]. Our findings demonstrate that HSF2BP promotes COX6A1 expression and mitochondrial complex IV activity, thereby enhancing fatty acid β-oxidation, reducing ROS accumulation, and alleviating ER stress. This suggests that targeting the HSF2BP–COX6A1 axis may represent a promising approach for MASLD intervention.

SUMOylation, as a form of post-translational protein modification, is involved in a variety of important physiological and biochemical reactions in eukaryotic cells. For example, SUMOylation can regulate interactions between proteins, the transport of proteins between the nucleus and cytoplasm, protein localization, and transcriptional activity, and it can also antagonize ubiquitination [45–48]. In this study, we identified that the SUMOylation of HSF2BP is instrumental in its lipid-regulating capabilities. Upon the onset of hepatic steatosis, the SUMOylation levels within hepatocytes diminish, correlating with a decrease in HSF2BP SUMOylation. This de-SUMOylation event prompts a relocation of HSF2BP from the nucleus to the cytoplasm, thereby altering its protein activity and neutralizing its influence on mitochondrial and lipid homeostasis. Consequently, even though HSF2BP levels surge following the development of fatty liver disease, its lipid-regulating properties are compromised due to the reduction in its SUMOylation. In our study, we observed that the SUMOylation of HSF2BP redirects its cellular localization from the cytoplasm back to the nucleus. Prior research from our group on HSF2BP in the context of acute liver injury models had noted a similar nuclear translocation of HSF2BP from the cytoplasm [17]. However, the mechanisms driving this cellular relocation were not elucidated in the previous study. Our current findings suggest that this shift in cellular distribution is likely a consequence of changes in HSF2BP’s SUMOylation status.

Growing evidence indicates a strong correlation between SUMOylation and the progression of liver diseases, including MASLD [24, 25, 49–51]. Consistently, our study also highlights the critical role of hepatic SUMOylation in the pathogenesis and potential treatment of MASLD. Pharmacological inhibition of SUMOylation using TAK-981, a novel SUMO E1 inhibitor, completely abolished the protective effects of HSF2BP overexpression in both cellular and mouse models of fatty liver, underscoring the necessity of SUMOylation for HSF2BP-mediated metabolic regulation. Conversely, global activation of SUMOylation—achieved either through genetic overexpression of UBC9 or pharmacological treatment with the small-molecule SUMO activator N106—significantly alleviated steatosis in HFD-induced MASLD models. Notably, these beneficial effects were markedly—but not entirely—attenuated in HSF2BP-deficient mice, suggesting that while HSF2BP is a major downstream effector of SUMOylation in regulating lipid metabolism, additional SUMOylated targets may also contribute to the improvement of hepatic steatosis. This observation not only reinforces the functional importance of HSF2BP SUMOylation in metabolic regulation but also opens avenues for uncovering other SUMO-dependent factors involved in liver lipid homeostasis.

Finally, although our co-immunoprecipitation (IP) and immunofluorescence staining experiments preliminarily demonstrated that the K222 residue of HSF2BP is a critical site for its SUMOylation, we also observed that mutation of the K222 site significantly reduced but did not completely abolish the SUMOylation band of HSF2BP around 75 kDa. Moreover, in immunofluorescence staining, although mutation at K222 caused a marked change in the subcellular localization of HSF2BP (from the nucleus to the cytoplasm), a portion of the protein remained in the nucleus. Collectively, these findings suggest that in addition to K222, there may be other unknown SUMOylation sites on HSF2BP. This might partially explain why the molecular weight of the SUMOylated HSF2BP band is approximately 40 kDa higher than its unmodified form. For future studies on HSF2BP, mass spectrometry analysis to identify its SUMOylation sites represents a worthwhile direction.

To summarize, our findings have established that HSF2BP is capable of modulating mitochondrial function in the liver, mitigating ER stress, and ameliorating the accumulation of lipids in MASLD. Importantly, the efficacy of HSF2BP in these processes is contingent upon its SUMOylation status. HSF2BP SUMOylation promoted its nuclear translocation, triggering an upregulation of COX6A1, a key element of the mitochondrial respiratory chain. This discovery offers valuable insights and potential therapeutic targets for the management and study of fatty liver disease moving forward.

## Methods

Detailed protocols are available in Supplementary Methods.

### Sex as a biological variable

Only male mice were used in this study to reduce variability in metabolic phenotypes. However, the findings are expected to be broadly applicable to both sexes.

### Animal studies

All animal experiments were performed using male C57BL/6J mice (6–8 weeks old, 18–24 g, free of overt pathology), housed under specific pathogen-free conditions at the Experimental Animal Center of Xi’an Jiaotong University. Mice were maintained on a 12-h light/dark cycle at 22 °C with ad libitum access to food and water. All procedures conformed to the Guidelines for the Care and Use of Laboratory Animals and were approved by the Institutional Animal Care and Use Committee (IACUC) of Xi’an Jiaotong University (Protocol Nos. XJTUAE2021-730 and 2025-563). Euthanasia was performed under isoflurane anesthesia to minimize suffering.

Liver-specific HSF2BP-TG and HSF2BP-HKO mice were generated using CRISPR-Cas9-mediated genome editing, as previously described [16]. For rescue experiments, UBC9 adenovirus (GeneChem Co., Ltd., Shanghai) or negative control virus was administered via tail vein injection. Liver tissues were collected 2 weeks post-injection for validation and downstream analyses.

### Animal experiment

To investigate the physiological role and SUMOylation dependency of HSF2BP in hepatic steatosis, three sets of in vivo experiments were performed using HSF2BP transgenic (TG), knockout (HKO), and appropriate littermate mice under various dietary and pharmacological conditions. Sample sizes (*n* ≥ 6) for animal experiments were determined based on prior experience with similar MASLD models and were consistent with those commonly used in the field to detect biologically meaningful differences in hepatic steatosis and mitochondrial function. In our previous studies using HSF2BP TG and HKO mouse models [16, 17], these group sizes were sufficient to detect robust and reproducible phenotypic differences between genotypes and treatments. Formal a priori statistical power calculations were not performed; however, the observed effect sizes were large and consistent, and statistical significance was achieved using appropriate tests as described in the Statistical Methods section. Randomization was performed using random number generation to minimize group selection bias. Investigators were not involved in outcome assessment during group allocation. No animals were excluded unless predefined technical issues occurred.

### 1. Functional role of HSF2BP in diet-induced hepatic steatosis

Eight-week-old male HSF2BP-TG and HSF2BP-HKO mice, along with their respective NTG and FLOX littermates, were fed either a high-fat diet (HFD, 60% kcal fat, D12492; Research Diets) or standard chow diet (CD) for 16 weeks. Metabolic and histological assessments were conducted at the endpoint to evaluate the impact of HSF2BP modulation on hepatic lipid accumulation, mitochondrial function, and oxidative stress.

### 2. Requirement of SUMOylation for HSF2BP-mediated protection

To investigate whether the protective effects of HSF2BP are SUMOylation-dependent, HSF2BP-TG and NTG mice were fed an HFD or CD for 12 weeks. From week 8 to 12, mice received either TAK-981 (a SUMOylation inhibitor, 7.5 mg/kg, tail vein injection every 3 days) or Vehicle control. Mice were sacrificed 24 h after the final treatment. The impact of SUMOylation inhibition on HSF2BP-mediated hepatoprotection was evaluated through histological and molecular assessments.

### 3. Testing whether SUMOylation-induced protection requires HSF2BP

To determine whether the beneficial effects of enhancing hepatic SUMOylation depend on HSF2BP, we performed both genetic and pharmacological SUMOylation activation in HSF2BP-HKO and FLOX control mice.

For genetic activation, 6-week-old mice received tail vein injections of either AAV8-UBC9 (80 µL, 5 × 10¹³ VG/mL; GeneChem) or control AAV. Two weeks post-injection, mice were fed HFD or CD for 12 weeks. For pharmacological activation, wild-type or HSF2BP-HKO mice received N106 (10 mg/kg/day, i.p.) from week 8 to 12 of HFD feeding.

Mice were sacrificed 24 h after the final treatment. The extent of hepatic lipid accumulation, mitochondrial integrity, and oxidative stress was analyzed.

### Statistics

All data are shown as mean ± SEM. F-test was used to determine the equality of variance. Student’s t-test or Mann-Whitney U test was used to compare the differences between two groups. One-way ANOVA or two-way ANOVA followed by Tukey’s or Šídák’s multiple comparison test was performed to analyze multiple groups. Statistical analysis was performed using GraphPad Prism. Statistical significance is represented as follows: ∗*p* < 0.05, ∗∗*p* < 0.01, ∗∗∗*p* < 0.001, and ns indicates no significance.

### Ethics statements

All human studies complied with ethical standards of the First Affiliated Hospital of Xi’an Jiaotong University (IRB approval number: LLSBPJ-2025-308). Informed consent was obtained from all subjects. All animals received human care, and the study protocols complied with the guidelines of animal care in experiments of the First Affiliated Hospital of Xi’an Jiaotong University (IACUC protocol: XJTUAE2021-730 and 2025-563).

## Supplementary information

Supplementary Figures and Legends

Supplementary Materials and Methods

Uncropped Western Blot

Reproducibility checklist

## Data Availability

All data are available in the main text or in the Supplementary Material.
