# Supplementary material for: SUMOylation-driven nuclear translocation of HSF2BP alleviates MASLD via COX6A1-dependent mitochondrial reprogramming
Source: Cell Death Dis. 2026 Jun 8;17(1):718. doi: 10.1038/s41419-026-08935-3 (PMC13469112; doi:10.1038/s41419-026-08935-3)
Supplement: Supplementary file 1 [file 41419_2026_8935_MOESM4_ESM.pdf]

Corresponding Author Name: Rongqian Wu
Manuscript Number: CDDIS-25-5878

Reporting Summary

Springer Nature wishes to improve the reproducibility of the work that we publish. This checklist is used to ensure good reporting standards and to improve the reproducibility. Please respond completely to all questions relevant to your manuscript. For more information, please read the journal's Guide to Authors.

☑ **Check here to confirm that the following information is available in the Material & Methods section:**

- **The exact sample size (*n*)** for each experimental group/condition, given as a number, not a range
- **A description of the sample collection** allowing the reader to understand whether the samples represent technical or biological replicates (including how many animals, litters, culture, etc.)
- **A statement of how many times the experiment shown was replicated in the laboratory**
- **Definitions of statistical methods and measures**: For small sample sizes (n<5) descriptive statistics are not appropriate, instead plot individual data points
    - Very common tests, such as *t*-test, simple χ^2 tests, Wilcoxon and Mann-Whitney tests, can be unambiguously identified by name only, but more complex techniques should be described in the methods section
    - Are tests one-sided or two-sided?
    - Are there adjustments for multiple comparisons?
    - **Statistical test results**, e.g., ***P* values**
    - Definition of **'center values'** as **median or mean**;
    - Definition of **error bars as s.d. or s.e.m. or c.i.**

Please ensure that the answers to the following questions are reported in the manuscript itself. We encourage you to include a specific subsection in the methods section for statistics, reagents and animal models. Below, provide the page number or section and paragraph number.

Statistics and general methods

	**Reported in section/paragraph or page #**
1. How was the sample size chosen to ensure adequate power to detect a pre-specified effect size? (Give section/paragraph or page #)	In the 'Animal experiment' section on page 22.
For animal studies, include a statement about sample size estimate even if no statistical methods were used.	As described in the 'Animal experiment' section on page 22, sample sizes were chosen based on prior experience and published studies using similar experimental models.
2. Describe inclusion/exclusion criteria if samples or animals were excluded from the analysis. Were the criteria pre-established? (Give section/paragraph or page #)	Detailed exclusion criteria for human samples are provided in the 'Human liver samples' section of the Supplementary Materials and Methods, and the animal experiments are described in the Animal Studies section on page 22.
3. If a method of randomization was used to determine how samples/animals were allocated to experimental groups and processed, describe it. (Give section/paragraph or page #)	In the 'Animal experiment' section on page 22.
For animal studies, include a statement about randomization even if no randomization was used.	Yes, we have provided a detailed description in the manuscript.

4. If the investigator was blinded to the group allocation during the experiment and/or when assessing the outcome, state the extent of blinding. (Give section/paragraph or page #)	In the 'Animal experiment' section on page 22.
For animal studies, include a statement about blinding even if no blinding was done.	Yes, we have provided a detailed description in the manuscript.
5. For every figure, are statistical tests justified as appropriate?	Yes, the statistical methods for this study are described in detail in the 'Statistical Analysis"'section on page 24 of the manuscript.
Do the data meet the assumptions of the tests (e.g., normal distribution)?	Yes, these details are summarized in the 'Statistical Analysis' section of the manuscript (Page 24)
Is there an estimate of variation within each group of data?	Yes, these details are summarized in the 'Statistical Analysis' section of the manuscript (Page 24)
Is the variance similar between the groups that are being statistically compared? (Give section/paragraph or page #)	Yes, these details are summarized in the 'Statistical Analysis' section of the manuscript (Page 24)

Reagents	Reported in section/paragraph or page #
6. Report the source of antibodies (vendor and catalog number)	All antibodies employed in this study are described in the 'Key Resources Table' , which is included in the Supplementary Materials and Methods file.
7. Identify the source of cell lines and report if they were recently authenticated (e.g., by STR profiling) and tested for mycoplasma contamination	All cell lines in this study are described in the 'Key Resources Table' , which is included in the Supplementary Materials and Methods file.

Animal Models	Reported in section/paragraph or page #
8. Report species, strain, sex and age of animals	In the 'Sex as a biological variable' and 'Animal studies' sections on Page 21.
9. For experiments involving live vertebrates, include a statement of compliance with ethical regulations and identify the committee(s) approving the experiments.	Yes, animal ethics approval has been provided in the 'Animal studies' section on Page 21.

10. We recommend consulting the ARRIVE guidelines (*PLoS Biol.* **8**(6), e1000412,2010) to ensure that other relevant aspects of animal studies are adequately reported.

Human subjects

	Reported in section/paragraph or page #
11. Identify the committee(s) approving the study protocol.	Yes, the ethics approval for human specimens has been described in the 'Human liver samples' section of the Supplementary Materials and Methods.
12. Include a statement confirming that informed consent was obtained from all subjects.	Yes, we confirm that informed consent was obtained from all participants, and this has been described in the 'Human liver samples' section of the Supplementary Materials and Methods.
13. For publication of patient photos, include a statement confirming that consent to publish was obtained.	No photographs of patients were published in this study.
14. Report the clinical trial registration number (at ClinicalTrials.gov or equivalent).	No clinical trials were conducted as part of this research.

15. For phase II and III randomized controlled trials, please refer to the CONSORT statement and submit the CONSORT checklist with your submission.

16. For tumor marker prognostic studies, we recommend that you follow the REMARK reporting guidelines.

Data deposition

	Reported in section/paragraph or page #
17. Provide accession codes for deposited data. Data deposition in a public repository is mandatory for:<br>a. Protein, DNA and RNA sequences<br>b. Macromolecular structures<br>c. Crystallographic data for small molecules<br>d. Microarray data	The deposition and availability of experimental data, including DNA and RNA sequences, genetic constructs, and proteomics datasets, have been detailed in the Key Resources Table within the Supplementary Materials and Methods.

Deposition is strongly recommended for many other datasets for which structured public repositories exist; more details on our data policy are available in the Guide to Authors. We encourage the provision of other source data in supplementary information or in unstructured repositories such as Figshare and Dryad. We encourage publication of Data Descriptors (see Scientific Data) to maximize data reuse.

18. If computer code was used to generate results that are central to the paper's conclusions, include a statement in the Methods section under "**Code availability**" to indicate whether and how the code can be accessed. Include version information as necessary and any restrictions on availability.	No relevant computer code was involved in the experiments.
