# Supplementary material for: SUMOylation-driven nuclear translocation of HSF2BP alleviates MASLD via COX6A1-dependent mitochondrial reprogramming
Source: Cell Death Dis. 2026 Jun 8;17(1):718. doi: 10.1038/s41419-026-08935-3 (PMC13469112; doi:10.1038/s41419-026-08935-3)
Supplement: Supplementary file 2 [file 41419_2026_8935_MOESM1_ESM.docx]

**Supplemental Figures and legends**

**Figure S1. Validation of HSF2BP overexpression and knockout both in vitro and in vivo.**

**A&B,** Verification of HSF2BP overexpression in HepG2 cells by Western blot (WB) (n=6/group); **C&D,** Verification of HSF2BP knockout in HepG2 cells by WB (n=8/group); **E&F,** WB validation of HSF2BP protein in liver specimens from HSF2BP-overexpressing mice (n=8/group); **G&H,** WB validation of HSF2BP protein in liver specimens from hepatocyte-specific HSF2BP knockout mice (n=6-8/group).

Data are presented as mean ± SEM. ^∗∗∗^*p* < 0.001.

**Figure S2. Knockout of HSF2BP exacerbates lipid deposition and oxidative damage in hepatocytes.**

**A&B,** Changes in body weight (A) and IPGTT analysis (B) of HSF2BP-HKO and FLOX groups of C57 mice fed with CD or HFD for 16 weeks (n=6/group); * (CD vs. HFD); ^#^ (NTG-HFD vs. TG-HFD); **C,** Changes in hepatic triglyceride/total cholesterol content of HSF2BP-HKO and FLOX groups (n=6/group); **D&E,** Western blot analysis of ADFP protein expression levels in livers of C57 FLOX and HSF2BP-HKO mice (n=6/group); **F,** The liver phenotype of C57 mice in the FLOX and HSF2BP-HKO groups fed with HFD for 16 weeks; **G,** HE and Oil Red O staining of livers from C57 FLOX and HSF2BP-HKO mice fed HFD for 16 weeks; **H,** qPCR analysis of mRNA expression levels of hepatic β-oxidation related genes (ACOX1, COX6A1, CPT1a, ACADM, ACADS, FABP1, ABCD1) in C57 FLOX and HSF2BP-HKO mice (n=6/group); **I&J,** WB analysis of mitochondrial-related proteins (TFAM, MT-ND2, MT-ND3) expression levels in livers of C57 FLOX and HSF2BP-HKO mice (n=6/group); **K,** The expression level of mitochondrial marker MT-ND1 mRNA in the liver of HSF2BP-HKO and FLOX mice (n=6/group); **L,** Representative images of mitochondrial Tracker staining in liver tissues of C57 FLOX and HSF2BP-HKO mice fed HFD for 16 weeks.

Data are presented as mean ± SEM. ^∗^*p* < 0.05, ^∗∗^*p* < 0.01, ^∗∗∗^*p* < 0.001; ^#^*p* < 0.05, ^###^*p* < 0.001;

**Figure S3. HSF2BP is associated with endoplasmic reticulum stress and mitochondrial function in hepatocytes.**

**A&B,** WB analysis of ER stress-related proteins (BIP, IRE1a, XBP1s, CHOP) in HSF2BP-TG mice vs. NTG mouse livers (n=6/group); **C&D,** WB analysis of SOD2 protein expression levels in livers of C57 NTG and HSF2BP-TG mice (n=6/group); **E,** Representative images of DHE staining in liver tissues of C57 NTG and HSF2BP-TG mice fed HFD for 16 weeks; **F-H,** Hepatic tissue levels of MDA (F), GSH (G) and SOD(H) in C57 NTG and HSF2BP-TG mice (n=6/group); **I&J,** WB analysis of ER stress-related proteins (BIP, IRE1a, XBP1s, CHOP) in HSF2BP-HKO vs. FLOX mouse livers (n=6/group); **K&L,** WB analysis of SOD2 protein expression levels in livers of C57 FLOX and HSF2BP-HKO mice (n=6/group); **M,** Representative images of DHE staining in liver tissues of C57 FLOX and HSF2BP-HKO mice fed HFD for 16 weeks; **N-P,** Hepatic tissue levels of MDA (I), GSH (J) and SOD(K) in C57 FLOX and HSF2BP-HKO mice (n=6/group);

Data are presented as mean ± SEM. ^∗^*p* < 0.05, ^∗∗^*p* < 0.01, ^∗∗∗^*p* < 0.001.

**Figure S4. Validation of HSF2BP SUMOylation and the effect of TAK-981.**

**A,** Expression levels of SUMO1 genes in the SteatoSITE database across clinically normal controls and MASLD patients at progressively advanced stages of disease severity (F0-F4). **B&C,** Western blot validation of SUMOylation inhibition in HepG2 cells treated with a gradient of TAK-981 concentrations for 48 hours (n=3/group); **D&E,** Western blot analysis of HSF2BP following immunoprecipitation with an HSF2BP antibody in cells overexpressing SUMO1 or SUMO2/3 (n=3/group)**.**

Data are presented as mean ± SEM.  ^∗^*p* < 0.05, ^∗∗∗^*p* < 0.001, ns: non-significance.

**Figure S5. The SUMOylated HSF2BP band appears at approximately 75 kDa.**

**A&B,** WB analysis of HSF2BP protein following immunoprecipitation with an HSF2BP antibody in HepG2 cells treated with either PA for 12h and TAK981 (T981) for 48h (n=3/group); **C&D,** IP with anti-HSF2BP antibody in NC and SUMO1&UBC9 overexpression groups, then IB with anti-SUMO1 antibody (n=3/group); **E&F,** IP with anti-SUMO1 antibody in NC and SUMO1&UBC9 overexpression groups, then IB with anti-HSF2BP antibody (n=3/group).

Data are presented as mean ± SEM.  ^∗∗∗^*p* < 0.001

**Figure S6. HSF2BP may be SUMOylated by SUMO1 at lysine 222.**

**A&B,** Prediction of SUMOylation sites on HSF2BP was performed using the online tools JASSA, SUMOplot, and GPS‑SUMO (A); High-probability candidate sites identified by consensus among the three tools are shown (B); **C,** Immunoprecipitation followed by WB analysis of HSF2BP in HepG2 cells overexpressing either the K75R or K222R HSF2BP point‑mutant plasmid; **D,** IP of SUMO1 or SUMO2/3 followed by WB for HSF2BP in HepG2 cells overexpressing the indicated HSF2BP point‑mutant plasmids (K75R or K222R). **E,** Representative images of immunofluorescence staining of HSF2BP following overexpression of wild-type and K222 mutant HSF2BP.

**Figure S7. SUMOylation of HSF2BP affects mitochondrial function.**

**A,** Representative images of ROS staining of HepG2 cell lines NC group and HSF2BP-NTG group after control, PA (12h), and T981 (48h) + PA (12h) treatments; **B,** Representative images of mitochondrial Tracker staining of HepG2 cell lines NC group and HSF2BP-TG group after control, PA (12h), and T981 (48h) + PA (12h) treatments; **C-H,** WB analysis of expression levels of ADFP, SOD2, and mitochondrial-related proteins (T-FAM, MT-ND2, MT-ND3) in HepG2 cell lines NC and HSF2BP-TG groups after PA (12h) and T981(48h) + PA (12h) treatments (n=3/group).

Data are presented as mean ± SEM. ^∗^p < 0.05, ^∗∗^*p* < 0.01, ^∗∗∗^*p* < 0.001.

**Figure S8. SUMO-activating agonist N106 and UBC9 overexpression both increase the SUMOylation of HSF2BP.**

**A,** WB analysis demonstrated that both UBC9 expression and SUMOylation levels in HepG2 cells were progressively reduced with prolonged PA treatment; **B,** Representative immunofluorescence co-staining images of HSF2BP (green) and UBC9 (red) following treatment with PA or PA+N106 in HepG2 cells; **C,** WB analysis of immunoprecipitation of HSF2BP following overexpression of UBC9 or N106 treatment in HepG2 cells, examining the SUMOylation status of HSF2BP (n=3/group).

Data are presented as mean ± SEM. ^∗^p < 0.05, ^∗∗^*p* < 0.01, ^∗∗∗^*p* < 0.001.

**Figure** **S9. Increased SUMOylation of HSF2BP attenuates lipid accumulation in hepatocytes under high-lipid conditions.**

**A,** Representative Oil Red O staining images of HepG2 cells in the NC, UBC9-TG, and UBC9 TG/HFS2BP-KO cell lines following 12 hours PA treatment; **B,** Representative Oil Red O staining images of HepG2 cells in the NC, N106, and N106/HFS2BP-knockout groups following 12 hours PA treatment; **C,** WB analysis revealed a marked reduction in both UBC9 expression and SUMOylation levels in the liver of C57BL/6 mice following HFD feeding (n=8/group); **D,** Representative images of HE and Oil Red O staining in liver tissues from C57BL/6 mice in the NC group, UBC9-AAV8 tail vein injection group, and UBC9-AAV8/HSF2BP-KO groups after 12 weeks of HFD feeding; **E,** Representative images of HE and Oil Red O staining of liver tissues from C57BL/6 mice in the NC, N106, and N106/HSF2BP-KO groups after 12 weeks of HFD feeding.

Data are presented as mean ± SEM.  ^∗∗∗^*p* < 0.001.

**Figure S10. Inhibition of HSF2BP SUMOylation abolishes its regulatory effects on COX6A1 and mitochondrial complex IV.**

**A,** Expression levels of the COX6A1 gene in the SteatoSITE database across clinically normal controls and MASLD patients at progressively advanced stages of disease severity (F0-F4); **B&C,** WB analysis of mitochondrial Complex proteins in normal human liver and MASLD patient liver tissues (n=8/group); **D&E,** WB analysis of COX6A1 expression levels in HepG2 NC cell lines and HSF2BP-TG cell lines after PA (12h) and T981 (48h) +PA (12h) treatments (n=3/group); **F&G,** WB analysis of COX6A1 expression levels in NTG group, HSF2BP-TG group, and HSF2BP-TG+T981 group mice after 12 weeks of HFD diet feeding (n=6/group); **H&I,** WB analysis of mitochondrial complex-related protein expression levels in liver tissue of C57 mice NTG, HSF2BP-TG, and HSF2BP-TG+T981 groups fed with HFD diet for 12 weeks (n=6/group).

Data are presented as mean ± SEM. ∗p < 0.05, ^∗∗^*p* < 0.01, ^∗∗∗^*p* < 0.001.

**Figure S11. COX6A1 influences lipid deposition and mitochondrial function.**

**A&B,** WB detection of expression levels of mitochondrial complex-related proteins in HepG2 cell lines COX6A1-TG group and NC group after control and PA (12h) treatments (n=3/group); **C&D,** OCR analysis of HepG2 cell lines COX6A1 overexpression group and its control group with palmitic acid substrate. Substrates include palmitic acid, sequentially added Eto/control, Oligo, FCCP, Rot/AA (n=5-6/group); **E,** Representative image of Oil Red O staining of HepG2 cell lines COX6A1-TG group and NC group after control and PA (12h) treatments; **F&G,** WB analysis of expression levels of ADFP, SOD2, and mitochondrial-related proteins (TFAM, MT-ND2, MT-ND3, COX6A1) in HepG2 cell lines COX6A1-TG group and NC group after control and PA (12h) treatments (n=3/group).

Data are presented as mean ± SEM. ∗p < 0.05, ^∗∗^*p* < 0.01, ^∗∗∗^*p* < 0.001.
