# Supplementary material for: SUMOylation-driven nuclear translocation of HSF2BP alleviates MASLD via COX6A1-dependent mitochondrial reprogramming
Source: Cell Death Dis. 2026 Jun 8;17(1):718. doi: 10.1038/s41419-026-08935-3 (PMC13469112; doi:10.1038/s41419-026-08935-3)
Supplement: Supplementary file 3 [file 41419_2026_8935_MOESM2_ESM.docx]

**Supplementary Materials and Methods:**

**Human liver samples**

Liver specimens were obtained from patients undergoing hepatic hemangioma resection with concomitant metabolic associated fatty liver disease (MASLD) at the First Affiliated Hospital of Xi'an Jiaotong University. Written informed consent was obtained from all patients prior to sample collection. Six patients without MASLD (hepatic hemangioma) from the same hospital were selected as normal controls. Exclusion criteria included pregnancy, severe liver diseases (cirrhosis, hepatocellular carcinoma, acute liver injury), organic digestive system disorders, autoimmune diseases, cancer, alcohol abuse (≥140 g per week for males or ≥70 g per week for females), continuous use of weight-loss drugs for more than one month, and gastrointestinal surgery. Human liver sections were blindly assessed by experienced pathologists using the Steatosis, Activity, and Fibrosis (SAF) scoring system. This study was approved by the Ethics Committee of the First Affiliated Hospital of Xi'an Jiaotong University (IRB approval number: LLSBPJ-2025-308).

**Glucose tolerance test**

An intraperitoneal glucose tolerance test (IPGTT) was performed to test the glucose tolerance of mice. After 8 h of fasting, mice were injected intraperitoneally with 2 g/kg glucose. Afterward, blood glucose was measured at 0, 30, 60, 90, and 120min using tail clippings.

**Measurement of oxidative stress**

Liver tissue homogenate was obtained and malonaldehyde (MDA) assay Kit (NanJing JianCheng Bioengineering Institute), superoxide dismutase (SOD) assay Kit (NanJing JianCheng Bioengineering Institute) and glutathione peroxidase activity (GSH-PX) assay Kit NanJing JianCheng Bioengineering Institute) were used for measuring the levels of liver MDA, SOD, GSH-PX according to the instructions of the manufacturer.

**Analysis of mitochondrial DNA (mtDNA) content**

mtDNA copy number was assessed by quantitative polymerase chain reaction (qPCR). The liver total DNA was isolated using Blood/Cell/tissue genomic DNA extraction kit (Tiangen Biotech Co., Ltd.) and mtDNA content was determined as mtDNA encoded NADH dehydrogenase-1 and normalized against the nuclear encoded POU class 5 homeobox 1 gene.

**Fluorescent microscopy of reactive oxygen species (ROS) in liver**

ROS in the liver were detected in situ using dihydroethidium (DHE) as previously described Cryostat sections of liver were cut into 10 μm thick and incubated with 5 (M DHE (Beyotime Institute of Biotechnology) at 37 °C for 30 min. DHE is oxidized by ROS to produce fluorescent ethidium that subsequently binds to nucleic acids, further staining the nucleus a bright fluorescence red. The red fluorescence from DHE was detected using a Leica TCS SP8 STED 3X fluorescence microscope. Three samples in each group were detected and the mean fluorescence density (MFD) was calculated as fluorescence intensity per unit area and assayed using Image-pro Plus 5.0 (Media Cybernetics, Corporation) by the method mentioned in our previous study

**Cellular reactive oxygen species (ROS) measurement.**

Dihydroethidium (DHE) dye (Sigma-aldrich,) was used for measuring intracellular accumulation of ROS levels. In brief, HepG2 cells were incubated with DHE dye for 30 min at a concentration of 3 μM. The staining cells were observed by a confocal microscope TCS SP8 STED 3× (Leica, Germany) with excitation wavelength 535 nm and emission wavelength 610 nm. The quantification of fluorescence intensity was performed using ImageJ2x software.

Fluorescent microscopy of ROS in liver

ROS in the liver were detected in situ using DHE as previously described Cryostat sections of liver were cut into 10 μm thick and incubated with 5um DHE at 37 °C for 30 min. DHE is oxidized by ROS to produce fluorescent ethidium that subsequently binds to nucleic acids, further staining the nucleus a bright fluorescence red. The red fluorescence from DHE was detected using a TCS SP8 STED 3× (Leica, Germany) fluorescence microscope. Three samples in each group were detected and the mean fluorescence density was calculated as fluorescence intensity per unit area and assayed using ImageJ2x software.

**Cellular MitoTracker Red fluorescence staining**

MitoTracker Red CMXRos dye (Solarbio Science & Technology Co., Ltd) was used for detection of mitochondria. HepG2 cells were incubated with MitoTracker Red CMXRos dye for 20 min at a concentration of 200 nM. The staining cells were observed by a confocal microscope with excitation wavelength 579 nm and emission wavelength 599 nm. The quantification of fluorescence intensity was performed using ImageJ2x software.

**Mitochondrial membrane potential (∆Ψm)**

The macrophages were stained with JC-1 following the manufacturer’s instructions of the JC-1 staining assay kit ( Beyotime Institute of Biotechnology). In brief, after interventions, cultured HepG2 cells were washed by 1 × PBS 3 times and stained with JC-1 staining solution for 20 min at 37 ℃. The absorption of monomer (fluorescence excitation set at 490 nm; fluorescence excitation set at 530 nm) and J-aggregates (fluorescence excitation 525 nm; fluorescence excitation 590 nm) was detected by Confocal microscope TCS SP8 STED 3× (Leica, Germany). The mitochondrial membrane potential difference between groups was reflected by the JC-1 ratio. The quantification of fluorescence intensity was performed using ImageJ2x software.

**Overexpressing lentivirus preparation and transfection**

HSF2BP, COX6A1 and UBC9 lentiviral overexpression vectors were synthesized by Genechem Corporation. Cells with target protein stable overexpression were obtained by lentivirus infection. HepG2 cells were seeded into 24-well plates at a density of 5×104 cells/well in 1 mL culture medium. The cells were cultured for 18 to 24h before being infected with lentiviruses. The virus-infected HepG2 cells were cultured for 3 days and the medium was exchanged every other day. The lentivirus used in this experiment, including target protein overexpressing lentivirus and the negative control lentivirus, were constructed by Genepharma Co., Ltd. HepG2 cells were infected with the virus in the presence of 2 μg/ml polybrene and selected with puromycin (2 μg/ml) for 2 weeks. After two rounds of infection, qRT-PCR and Western blot analyses were used for verification.

**siRNA transfection**

Small interfering RNAs duplexes for HSF2BP and UBC9 were provided by Genepharma. Transfection steps were following the manufacture's protocols. The day before transfection, 2-4×105 cells were seeded onto a 6-well plate and the cells reached 60-70% confluency the next day. In 250μl MEM (Biological Industries), 100pmol siRNA was added and gently mixed, followed by a 5 minutes incubation at room temperature. In another 250μl MEM, 5μl lipofectamine 3000 reagent (Thermo Scientific) was added and gently mixed, followed by a 5 minutes incubation at room temperature. The prepared siRNA and lipofectamine solutions were combined and gently mixed to form siRNA/lipofectamine complexes, which were then incubated at room temperature for 20 minutes. Subsequently, 500μl siRNA/lipofectamine complexes were added to a well containing 1.5ml culture medium, and the plate was gently rocked back and forth to ensure proper distribution. The cells were then incubated in a CO2 incubator at 37°C. The medium was replaced after 6-8 hours, and other post-transfection procedures were conducted 48 hours later.

**Extracellular flux analysis**

The oxygen consumption rate (OCR) was measured using a Seahorse XF Cell Mito Stress Test Kit (Agilent) with the Seahorse XFe24 Extracellular Flux Analyzer following the manufacturer's instructions. Briefly, HepG2 cells were isolated and seeded in XFe24 microplates at a density of 1 × 105 cells/well. Palmitic acid (PA, 200 μM) or normal medium incubate for 24 h. Before the assay, seahorse XF calibration solution (1 mL/well) was added to a utility plate covered with Hydro Booster and sensor cartridge, then placed overnight in a 37 °C CO2-free incubator with a hydration probe. The assay medium was prepared with DMEM medium, to which 1 mM pyruvate, 2 mM glutamine and 10 mM glucose were added. The cells were washed with assay medium twice and placed in a 37 °C CO2-free incubator for 1 h. Three compounds were injected into the microplate at a concentration of oligomycin (1.5 μM), carbonylcyanide-4-(trifluoromethoxy) phenylhydrazone (FCCP; 2.0 μM), and a mixture of rotenone and antimycin A (0.5 μM) according to the protocol. Then, the microplate was loaded, and the experimental program was started in the XFe24 Analyzer. After the assay, OCR was analyzed with protein content homogenized to exclude the cell number difference between wells. The basal mitochondrial respiration, ATP-linked oxygen consumption rate, maximal respiration, and spare respiratory capacity were calculated.

**Hematoxylin & eosin staining**

The liver tissues fixed in paraformaldehyde (4%) for 48 h were embedded in paraffin. Five-millimeter slices were cut for Hematoxylin & Eosin staining. An established grading system was used to evaluate the liver histological score. In brief, the liver histological score was the sum of the individual scores of steatosis (0-3), ballooning (0-2), and lobular inflammation (0-3) according to the MASLD scoring system. The liver necrosis area was evaluated by Image J software.

**Oil Red O staining**

HepG2 cells were fixed by 1 mL of 10% neutralized formalin for 10 min at room temperature. Following fixation, cells were washed with deionized water, and then incubated with 60% isopropanol for 5 min. Cells were stained with Oil Red O working solution (2 g/L Oil red O, 60% isopropanol, 40% H2O) for 10 minutes at room temperature, and then washed by deionized water until the water was clear. For tissues, mice were perfused with 4% Paraformaldehyde, post-fixed in 4% Paraformaldehyde for another 30 min, and put in 20% sucrose at 4°C for dehydration. Tissues were embedded in optimal cutting temperature medium for section. Frozen tissue sections were then stained with oil red o stain kit according to manufacturer’s instruction (Solarbio).

**Mitochondria isolation**

Cell mitochondria were isolated from HepG2 cells using Mitochondria Isolation Kit (Nanjing Jiancheng). HepG2 cells (2-4×106) were suspended in prechilled homogenization buffer, on ice for 2 min, and homogenized for 45 strokes using a Dounce homogenizer. The homogenate was added buffer C, then centrifuged at 4 °C at 800×g for 10 min and the supernatant was centrifuged at 4 °C at 12,000×g for 15 min. The supernatant was collected as cytosol fraction and the mitochondria were in the pellet. To obtain a more purified fraction of mitochondria, the pellet was washed again. The purity of the mitochondria was validated by Western blotting.

**Co-immunoprecipitation (IP) assay**

HepG2 cells were transfected with HSF2BP lentivirus or negative control. Cell lysates were collected using RIPA agent (Beyotime), and 1 mg of total protein lysates was used for IP from each sample. Then, the samples were incubated with 1 μg primary antibodies for 4 h at 4 °C with gentle agitation. Afterward, 10 μL of Protein A/G PLUS-Agarose (Santa Cruz) for each sample was added and incubated overnight at 4 °C. The mixture was washed 5 times, and protein was eluted with immunoprecipitation buffer for subsequent immunoblotting as described above.

**Primary hepatocyte isolation**

Primary hepatocytes were isolated from NTG, HSF2BP-TG, FLOX,HSF2BP-HKO male mice by a 2-step collagenase perfusion method as described previously. In brief, we clamped the supradiaphragmatic inferior vena cava of mice, and infused 30 mL perfusion solution (calcium and magnesium-free HBSS + 0.5 mM EGTA) through inferior vena cava followed by 30 mL collagenase IV solution (HBSS containing calcium and magnesium + 0.5 mg/mL collagenase IV + 15 μg/mL DNase I). Cells were then gently dissociated from digested livers, passed through 100 μm strainer, and centrifuged at 50 x g for 3 min at 4 °C. The supernatant was removed and hepatocyte pellets were resuspended in HBSS and loaded carefully onto a 45% Percoll gradient. Centrifuge at 1300 x g for 25 min at 4 °C. The pellets were resuspended and centrifuged at 650 x g for 7 min at 4 °C. The supernatant was removed and hepatocyte pellets were collected and finally cultured at a concentration of 5 x 105 cells/mL in DMEM medium supplemented with 10% heat-inactivated fetal bovine serum and 1% penicillin-streptomycin.

**TUNEL staining**

Liver injury was determined using the TUNEL kit according to the manufacturer’s instructions. Briefly, paraffin-embedded liver sections were routinely dewaxed and hydrated, and then permeabilized by proteinase K. After being incubated in 3% H2O2 for 10 min at room temperature, the slides were then covered with the TdT reaction solution for 60 min in humidified chamber at 37 °C. Finally, the sections were visualized with DAB. Image-Pro Plus 6.0 software was used to quantify the positive area.

**Immunohistochemistry staining**

Immunohistochemistry of mouse HSF2BP, COX6A1, SUMO1, SUMO2, UBC9 expression was performed on paraffin-embedded liver sections using anti- HSF2BP, COX6A1, SUMO1, SUMO2, UBC9 antibody. In brief, liver sections were routinely dewaxed and hydrated. After antigen retrieval, the sections were covered in 3% H2O2 to quench the endogenous peroxidase activity, and blocked with 5% BSA for 30 min at room temperature. The slides were then incubated with primary antibody overnight at 4 °C in a wet box. Horseradish peroxidase (HRP)-conjugated rabbit anti-rat IgG was used as the secondary antibody. The staining score was calculated by the sum of staining intensity (0, negative; 1, mild; 2, moderate; and 3, severe), and percentage of positive cells (0, negative; 1, 1–25%; 2, 26–50%; 3, 51–75%; and 4, 76–100%), ranging from 0-7sd.

**Immunofluorescence staining**

HepG2 cells were immobilized with 4% paraformaldehyde. Then the sections were permeabilized with 0.2% Triton X-100 for 15 min and blocked with 5% BSA for 30 min at room temperature. Subsequently, HepG2 cells were incubated with primary mouse HSF2BP (1:50 dilution) antibody and rabbit UBC9 (1:50 dilution) antibody overnight at 4°C. Then samples were incubated with Alexa Fluor 594–conjugated Goat Anti-Mouse IgG (1:200 dilution) and CoraLite488–conjugated Donkey Anti-Rabbit IgG (1:200 dilution) were incubated for 1 h at room temperature. DAPI was used to stain nuclei. The results were observed with a confocal microscope (TCS SP8 STED 3X, Leica, Germany).

**RNA extraction and quantitative real-time PCR**

Total RNA was extracted from liver tissues or hepatocytes or cultured cells by TRIzol reagent and reversely transcribed into cDNA using RevertAid First strand cDNA Synthesis Kit according to the manufacturer’s instructions. RT-qPCR was performed using SYBR Green. The primer sequences are listed in the key resources table. Relative mRNA expression was normalized to β-actin and calculated by the 2-ΔΔCt method.

**GO Annotation**

Gene Ontology (GO) analysis is a bioinformatics analysis method that can organically link the various information of genes and gene products (such as proteins) to provide statistical information. In proteomics projects, GO mainly has the following uses: 1. As a database of various information of proteins and genes; 2. Providing various information of proteins and genes and classifying proteins and genes according to the information; 3. As A tool that provides the most comprehensive annotation and classification services for all proteins in the project. GO analysis mainly includes three aspects: 1. Cellular Component: Refers to the specific component of the cell. In the GO system, it should the component of the larger element of the cell. For example, some cell structures in anatomy.(rough endoplasmic reticulum, nucleus, etc.), or a series of gene products, such as the basic structure of some complex components (ribosomes, protein dimers, etc.); 2. Molecular Function: Mainly describe the chemical activity of the molecule, such as the catalytic activity or binding activity at the molecular level; 3. Biological Process: A series of elements in the body executed a specific function in order, which is called the biological process. GO annotation is to annotate and analyze the identified proteins with eggnog-mapper software (v2.0). The software is based on the EggNOG database. The latest version is the 5th edition, covering 5,090 organisms (477 eukaryotes, 4445 representative bacteria and 168 archaebacteria) and 2502 virus genome-wide coding protein sequences. Extracting the GO ID from the results of each protein note, and then classified the protein according to Cellular Component, Molecular Function and Biological Process.

**KEGG Pathway Annotation.**

Kyoto Encyclopedia of Genes and Genomes (KEGG) can integrate the information known at present, such as pathways and related complexes (Pathway database), genes and gene products (Gene database), biological complexes and related reactions (Compound and Reaction database). The KEGG pathway mainly includes: metabolism, genetic information processing, environmental information processing, cellular processes, human diseases, drug development, and the like. We annotated the protein pathways based on the KEGG pathway database, and performed BLAST alignments (blastp, evalue≤1e-4) of the identified proteins. For the BLAST alignment results of each sequence, the alignment with the highest score (score) was selected to annotate.

**Functional Enrichment**

Enrichment of Gene Ontology analysis:GO annotations of proteins are divided into three broad categories: Biological Process, Cellular Component, and Molecular Function. Fisher’s exact test was used to analyze the significance of GO enrichment of differentially expressed proteins (using the identified protein as the background), and P value <0.05 were considered significant.

Enrichment of KEGG pathway analysis:Kyoto Encyclopedia of Genes and Genomes (KEGG) database was used for KEGG pathway enrichment analysis. Fisher’s exact test was used to analyze the significance of KEGG pathway enrichment of differentially expressed proteins (using the identified protein as the background) , and P value <0.05 were considered significant.

**Supplementary Table1 : Key resources table**

| REAGENT or RESOURCE | SOURCE | IDENTIFIER |
| --- | --- | --- |
| Antibodies | | |

| Anti-Heat Shock Factor 2 Binding | Abcam | ab126252 RRID: AB_11127293 |
| --- | --- | --- |
| Anti-Perilipin 2 | Abcam | ab108323 RRID: AB_10863476 |
| Anti-HSF2BP | Santa cruz | sc-130322 RRID: AB_2120598 |
| TFAM Polyclonal antibody | Proteintech | 22586-1-AP RRID: AB_11182588 |
| ND2 Polyclonal antibody | Proteintech | 19704-1-AP RRID: AB_10638920 |
| ND3 Polyclonal antibody | Proteintech | 29341-1-AP RRID: AB_3669683 |
| ATP5A1 Polyclonal antibody | Proteintech | 14676-1-AP RRID: AB_2061761 |
| MTCO2 Polyclonal antibody | Proteintech | 55070-1-AP RRID: AB_10859832 |
| UQCRC2 Polyclonal antibody | Proteintech | 14742-1-AP RRID: AB_2241442 |
| SDHB Polyclonal antibody | Proteintech | 10620-1-AP RRID: AB_2285522 |
| NDUFB8 Polyclonal antibody | Proteintech | 14794-1-AP RRID: AB_2150970 |
| SUMO1 Polyclonal antibody | Proteintech | 10329-1-AP RRID: AB_2286872 |
| SUMO2/3 Polyclonal antibody | Proteintech | 11251-1-AP RRID: AB_2198405 |
| Histone H3 Polyclonal antibody | Proteintech | 17168-1-AP RRID: AB_2716755 |
| SOD2 Polyclonal antibody | Proteintech | 24127-1-AP RRID: AB_2879437 |
| COX6A1 Polyclonal antibody | Proteintech | 11460-1-AP RRID: AB_2085445 |
| Anti-GRP78 BiP antibody | Abcam | ab21685 RRID: AB_2119834 |
| Anti-IRE1 (phospho S724) | Abcam | ab48187 RRID: AB_873899 |
| CHOP; GADD153 Polyclonal antibody | Proteintech | 15204-1-AP RRID: AB_2292610 |
| Anti-IRE1 | Abcam | ab37073 RRID: AB_775780 |
| XBP1S-specific Polyclonal antibody | Proteintech | 24868-1-AP RRID: AB_2879766 |
| Anti-UBE2I / UBC9 | Abcam | ab75854 RRID: AB_1310787 |
| 6*His, His-Tag Monoclonal antibody | Proteintech | 66005-1-Ig RRID: AB_11232599 |
| GAPDH Polyclonal antibody | Proteintech | 10494-1-AP RRID: AB_2263076 |
| HRP-conjugated Affinipure Goat Anti-Rabbit IgG(H+L) | Proteintech | SA00001-2 RRID: AB_2722564 |
| HRP-conjugated Affinipure Goat Anti-Mouse IgG(H+L) | Proteintech | SA00001-1 RRID: AB_2722565 |
| Rabbit IgG control Polyclonal antibody | Proteintech | 30000-0-AP RRID: AB_2819035 |
| Mouse IgG | Proteintech | B900620 RRID: AB_2883054 |

| Bacterial and virus strains | | |
| --- | --- | --- |
| Lentivirus (LV)-HSF2BP: pCMV-HSF2BP-3FLAG-puromycin | Genechem | N/A |
| LV-UBC9: pCMV-UBC9-6*his-puromycin | Genechem | N/A |
| LV-HSF2BP-RNAi: phU6-HSF2BPi-puromycin | Genechem | N/A |
| Adeno-Associated Virus (AAV) UBC9:AAV9-UBC9-pEGFP-N3 | Genechem | N/A |
| LV-COX6A1: pUbi-COX6A1-3FLAG-puromycin | Genechem | N/A |
| LV-COX6A1-RNAi: pUbi-COX6A1i-3FLAG-puromycin | Genechem | N/A |
| Overexpression plasmid HSF2BP (K75R)-Homo: CMV-MCS-HSF2BP (K75R)- SV40-Puromycin | Genechem | N/A |
| Overexpression plasmid HSF2BP (K222R)-Homo: CMV-MCS-HSF2BP (K222R) -SV40-Puromycin | Genechem | N/A |
| Biological samples |  |  |
| Liver samples from patients with MASLD and healthy controls |  | N/A |
| Chemicals, peptides, and recombinant proteins | | |

| MitoTracker Red CMXRos | Solarbio | M9940 |
| --- | --- | --- |
| Palmitic acid | Sigma-Aldrich | P5585 |
| Reactive Oxygen Species | Solarbio | CA1410 |
| mitochondrial membrane potential（JC-1） | Beyotime | C2003S |
| N106 | MCE | HY-110273 |
| Subasumstat (TAK-981） | Selleck | S8829 |
| Penicillin-streptomycin | Hyclone | SV30010 |
| Pancreatic enzymes | Hyclone | SH30042.01 |
| Tween-80 | Beyotime | ST2789-1L |
| DMSO | Sigma-Aldrich | 20-139 |
| Protein A/G PLUS-Agarose | Santa Cruz | sc-2003 |
| TRIzol Reagent | Sigma-Aldrich | T9424 |
| RevertAid Master Mix | Thermo Scientific | REF#M1631 |
| Fast SYBR Green Master Mix | Thermo Scientific | REF#06924204001 |
| lipo3000 | Thermo Scientific | L3000015 |
| MEM | Biological Industries | 01-025-1A |
| DMEM/HIGH GLUCOSE | Biological Industries | 01-052-1A |
| FBS | Biological Industries | 04-001-1A |
| Seahorse XF DMEM pH 7.4 | Agilent | 103575-100 |
| Seahorse XF 1.0 mol/L Glucose | Agilent | 103577-100 |
| Seahorse XF 100 mmol/L Pyruvic Acid | Agilent | 103578-100 |
| Seahorse XF 200 mmol/L Glutamine | Agilent | 103579-10 |

Critical commercial assays

| Triglyceride assay kit | Nanjing Jiancheng, Nanjing, China | A110-1-1 |
| --- | --- | --- |
| Total cholesterol assay kit | Nanjing Jiancheng, Nanjing, China | A111-1-1 |
| Superoxide Dismutase (SOD) assay kit | Nanjing Jiancheng, Nanjing, China | A001-3-2 |
| Malondialdehyde (MDA) assay kit | Nanjing Jiancheng, Nanjing, China | A003-1-2 |
| Reduced glutathione (GSH) assay kit | Nanjing Jiancheng, Nanjing, China | A006-2-1 |
| Mitochondria Isolation Kit | Nanjing Jiancheng, Nanjing, China | G006-1-1 |
| Xf Palmitate Oxidation Pressure Test Kit | Agilent | 103693-100 |
| Mitochondria respiratory chain complex IV assay kit | Nanjing Jiancheng, Nanjing, China | A089-4-1 |
| Blood/Cell/tissue genomic DNA extraction kit | TIANGEN | DP304-03 |
| BCA Protein Assay Kit | Beyotime | P0010 |
| ALT Assay Kit | Nanjing Jiancheng, Nanjing, China | C009-2-1 |
| AST Assay Kit | Nanjing Jiancheng, Nanjing, China | C010-2-1 |
| Oil Red O stain kit | Solarbio | G1262 |

Experimental models: Cell lines

| HepG2 | ATCC | HB-8065 |
| --- | --- | --- |
| Primary Mouse Hepatocytes |  | N/A |

| Oligonucleotides | | |
| --- | --- | --- |
| LV-siRNA targeting sequence: HSF2BP: CGACGGAAGTGATGCAAATCC | Genechem | N/A |
| LV-siRNA targeting sequence: COX6A1: CCAACTGGCTACGAAGATGAA | Genechem | N/A |
| siRNA targeting sequence: UBC9: GAGGCCUACACGAUUUACUTT | GenePharma | N/A |
| Primers for qPCR, see Table S1 |  |  |
| Software and algorithms | | |

| GraphPad Prism | Graphpad Software | N/A |
| --- | --- | --- |
| Image-Pro Plus 6.0 | Media Cybernetics Software | N/A |
| DAVID | www.davidbioinformatics.nih.gov | N/A |
| NovoSampler Pro | Agilent | N/A |
| SteatoSITE APP | shiny.igc.ed.ac.uk/SteatoSITE_gene_explorer | N/A |
| Las X 3.5.7 | Leica | N/A |
| BioRender | https://BioRender.com/ygb3dh8 | N/A |

| Deposited data | | |
| --- | --- | --- |
| The raw data of Proteomics | Mendeley Data | https://data.mendeley.com/preview/z7y9zdjppt?a=03b18056-8ba7-4329-a05c-ba798511564c |

| Other |  |
| --- | --- |

| Control Diet | Xietong Shengwu | 1010002 |
| --- | --- | --- |
| HFD Diet | Research Diets | D12492 |

**Supplementary Table2 : Primer sequences used in the study**

| **Primer for Quantitative reverse transcription PCR:** | |
| --- | --- |
| **Gene Symbol** | **Sequence of primers** |
| HSF2BP-F-mouse | ACCATGTGGCAGTGAATATCCCAGA |
| HSF2BP-R-mouse | CCAGGGAAGGGAAGGAGTCTTCTA |
| ABCD-F-human | CATCATCACTGCCACTGGCTAC |
| ABCD-R-human | GATCCGCTCAATGGCATCTG |
| ABCD1-F-mouse | AAGCAACATGGATGGACGAC |
| ABCD1-R-mouse | GGCTTGGTCAGGTTGGAATAAA |
| CPT1a-F-mouse | GGTAGATGTTTCGACAGGTGGTT |
| CPT1a-R-mouse | TCTGCGTTTATGCCTATCTTGCT |
| FABP1-F-mouse | CCAGGAGAACTTTGAGCCATTC |
| FABP1-R-mouse | GGGTCCATAGGTGATGGTGAG |
| ACADM-F-human | TGCAGATACTTGGAGGCAATGG |
| ACADM-Rhuman | CTTGTCAATGTGTTCACGGGCTAC |
| ACADS-F-human | AGAAGGAGTTGTTTCCCATTGCAG |
| ACADS-R-human | CAGGTAATCGAGGCCAGCAC |
| CPT1a-F-human | CTACTACACGGCCGATGTTACGA |
| CPT1a-Rhuman | GCATCTGCCCAGGAGTGTTC |
| FABP1-F-human | AGTACCAACTGCAGAGCCAGGA |
| FABP1-Rhuman | ACTTTGGACCCAGCGGTGA |
| SOD2-F-human | ACACTTACAAATTGCTGCTTGTCCA |
| SOD2-Rhuman | GTAGTAAGCGTGCTCCCACACATC |
| ACADM-F-mouse | TGATGTGGCGGCCATTAAGA |
| ACADM-R-mouse | GGGTTAGAACGTGCCAACAAGAA |
| ACADS-F-mouse | CTTTGCCGAGAAGGAGTTGGTC |
| ACADS-R-mouse | CCCGAGCTCACCCATCTTCTTA |
| SOD2-F-mouse | AATCTCAGTGCTCACTCGTGTCATC |
| SOD2-R-mouse | ATGCTGCCAGTCAGAAATCCAA |
| GAPDH-F-human | GCACCGTCAAGGCTGAGAAC |
| GAPDH-R-human | TGGTGAAGACGCCAGTGGA |
| GAPDH-F-mouse | AAATGGTGAAGGTCGGTGTGAAC |
| GAPDH-R-mouse | CAACAATCTCCACTTTGCCACTG |
| COX6A1-F-human | AGAGACCCGAGTTCATCGCCTA |
| COX6A1-R-human | TGCAGAGTAACGGTCCAAACCA |
| COX6A1-F-mouse | CTACCCTCACCTGCGCATCA |
| COX6A1-R-mouse | GTCCACACAGAGTAAGGGTCCAA |
| MT-ND1-F-mouse | GGATAGGTCCCAACAACCA |
| MT-ND1-R-mouse | GCTTGATCGTCCGCTACGGA |
